# Side-on coordination of boryl and borylene complexes to cationic coinage metal fragments†
†Electronic supplementary information (ESI) available: Experimental; details of X-ray crystallographic and computational studies. CCDC 1033284-1033294. For ESI and crystallographic data in CIF or other electronic format see DOI: 10.1039/c5sc00211g


**DOI:** 10.1039/c5sc00211g

**Published:** 2015-03-10

**Authors:** Holger Braunschweig, Krzysztof Radacki, Rong Shang

**Affiliations:** a Institut für Anorganische Chemie , Julius-Maximilians-Universität Würzburg , Am Hubland , 97074 Würzburg , Germany . Email: h.braunschweig@mail.uni-wuerzburg.de

## Abstract

The M-(η^2^-BMn) complex [(η^5^-C_5_H_5_)(OC)_2_Mn{μ-B(Cl)(*t*Bu)Au(PPh_3_)}] (**2**) can be functionalized *via* halide substitution reactions to afford isostructural complexes [(η^5^-C_5_H_5_)(OC)_2_Mn{μ-B(R)(*t*Bu)Au(PPh_3_)}] (R = Ph, CCPh and NCS). It also reacts with coinage metal complexes [MCl(PPh_3_)] (M = Au, Ag and Cu) in the presence of halide abstraction reagents to afford borylene-bridged heteromultinuclear complexes [{(η^5^-C_5_H_5_)(OC)_2_Mn}_2_{μ^2^-B(*t*Bu)}_2_M][BArx4] (M = Au, Ag and Cu; Ar^x^ = 3,5-C_6_H_3_Cl_2_, 3,5-C_6_H_3_(CF_3_)_2_). Experimental characterization as well as computational studies revealed that these complexes are best viewed as transition metal borylene complexes side-on coordinated to monovalent coinage metal cations, thus representing the first boron analogs of Stone's alkylidyne-bridged multinuclear complexes.

## Abstract 2

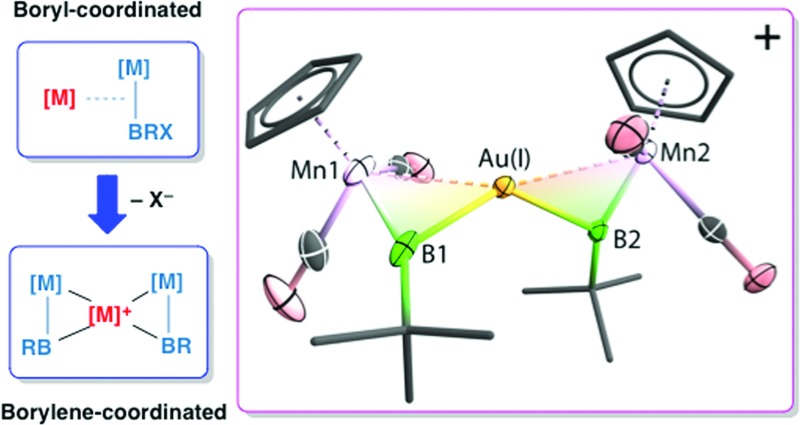
The M-(η^2^-BMn) complex [(η^5^-C_5_H_5_)(OC)_2_Mn{μ-B(Cl)(*t*Bu)Au(PPh_3_)}] (**2**) can be functionalized *via* halide substitution reactions to afford isostructural complexes [(η^5^-C_5_H_5_)(OC)_2_Mn{μ-B(R)(*t*Bu)Au(PPh_3_)}] (R = Ph, CCPh and NCS).

## Introduction

**Chart 1:**
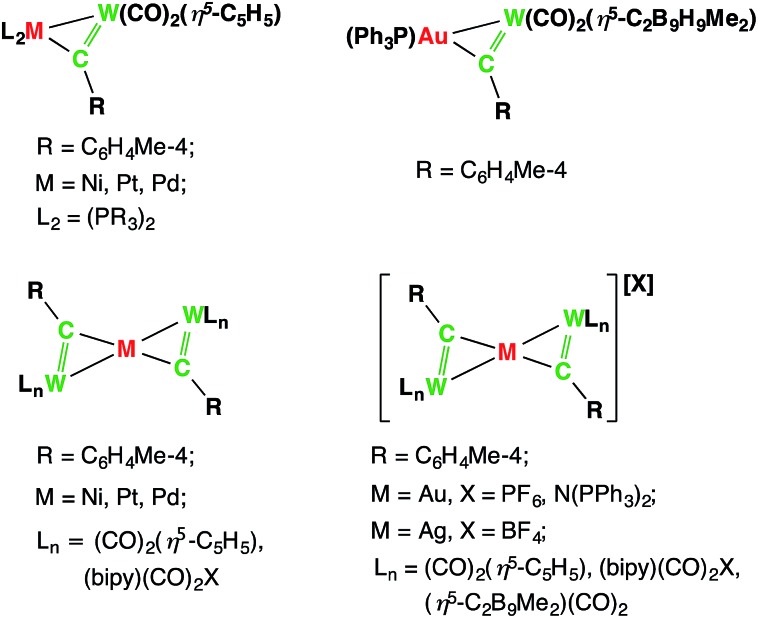
Selected examples of side-on-coordinated metal alkylidyne complexes.

The concepts of isoelectronicity and isolobality have proven to be immensely useful in the strategic design and construction of molecules, rationalizing bonding and reactivity by providing a short-cut to understanding the electronic structure of less well-known systems using well-established systems.^1^ One remarkable demonstration of their application can be found in Stone's studies of unsaturated systems containing metal–carbon multiple bonds, where the alkylidene and alkylidyne metal fragments were compared to their isolobal organic fragments CR_2_ and CR respectively to rationalize alkylidene- and alkylidyne-derived bi- and multi-metallic frameworks, such as those shown in Chart 1.^2^–^8^

**Chart 2:**
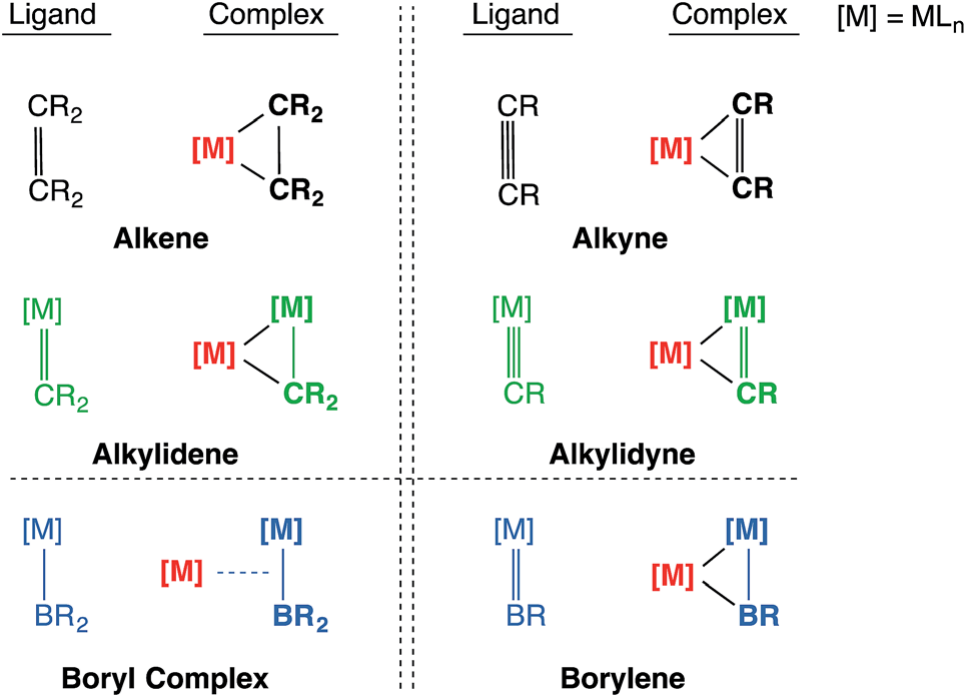
The isolobal analogy: alkene/alkyne, alkylidene/alkylidyne, boryl/borylene and their side-on coordination to a second transition metal center.

Transition metal boryl complexes, by virtue of their weak π-back bonding interactions, are isolobal with alkenes and alkylidenes in the same way that transition metal borylene complexes are isolobal with alkynes and alkylidyne complexes.^9^,^10^ Following the guidance of these concepts, one may postulate analogous molecular motifs by invoking the isolobal and isoelectronic fragment pairs alkylidene [CR_2_]/boryl [BR_2_]^–^ and alkylidyne [CR]^+^/borylene [BR] as shown in Chart 2. This study reports the first bisborylene coinage metal complexes [{(η^5^-C_5_H_5_)(OC)_2_Mn}_2_{μ^2^-B(*t*Bu)}_2_M][BArx4] (M = Au, Ag and Cu; Ar^x^ = C_6_H_3_Cl_2_, C_6_H_3_(CF_3_)_2_),^11^,^12^ which formed from halide-abstraction reactions of the recently reported σ-coordinated boryl complex [(η^5^-C_5_H_5_)(OC)_2_Mn{μ-B(Cl)(*t*Bu)}Au(PPh_3_)] (**2**).^13^

These complexes possess a distinctly different structural architecture to the relatively small library of multi- and di-nuclear borylene complexes, all of which have been described in terms of [BR] bridging.^10^,^12^,^14^–^21^ Among these, the [BR] ligands are commonly found to symmetrically bridge two metal centers, while only a few group 6 aminoborylenes have been shown to demonstrate a ‘semi-bridging’ motif of the borylene ligand B=N(SiMe_3_)_2_.^22^–^24^

This work also reports the first detailed computational study that treats the metal borylene moiety [M=B(R)] as a ligand, which reveals that it serves as a side-on π-ligand that resembles classical metal olefin or alkyne π-interactions and thus also their isolobal alkylidyne complexes (Chart 2).^25^ The syntheses, reactivity and bonding situations of these novel boryl- and borylene-coordinated coinage metal(I) complexes are herein reported.

## Results and discussion

**Scheme 1:**
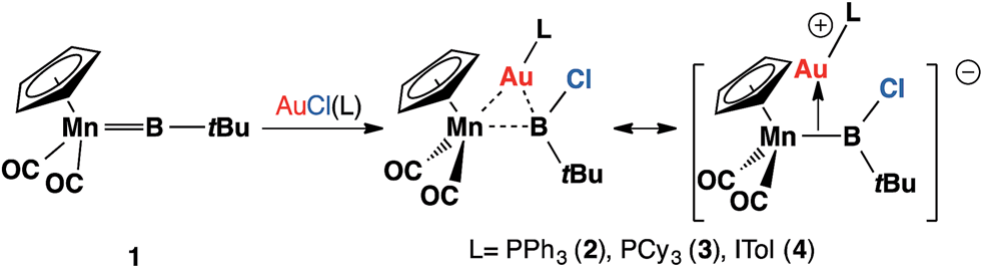
Recently-reported reaction of 1 with [AuCl(L)] affords heterodinuclear complexes [(η^5^-C_5_H_5_)(OC)_2_Mn{μ-B(Cl)(*t*Bu)}Au(L)] (L = PPh_3_, **2**; PCy_3_, **3**; ITol, **4**).

Recently, we have communicated that the complex [(η^5^-C_5_H_5_)(OC)_2_Mn=B*t*Bu] (**1**) reacted with gold(i) chloride complexes to form heterodinuclear complexes [(η^5^-C_5_H_5_)(OC)_2_Mn{μ-B(Cl)(*t*Bu)}Au(L)] (L = PPh_3_, **2**; PCy_3_, **3**; ITol, **4**; ITol = {C_3_N_2_H_2_(C_6_H_4_Me)_2_}), which are best viewed as σ-coordinated transition metal haloboryl complexes.^13^ Among these, the reaction with AuCl(PPh_3_) affords the best yield and therefore complex **2** has been used for subsequent studies (Scheme 1).

**Scheme 2:**
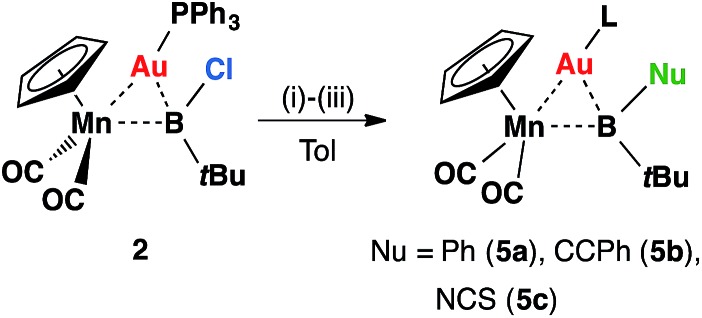
Formation of **5a–c***via* halide displacement reactions of **2**: (i) LiPh, (ii) LiCCPh, (iii) [NBu_4_][SCN].

Intuitively, the reactive boron–halogen bond in haloboryl complexes should be relatively straightforward to functionalize, yet in reality, examples of such a reaction remain rare. Most of these transformations introduce π-donating –OR and –NR_2_ substituents to the boryl ligand.^26^ Therefore, reactions of **2** with nucleophiles were investigated for halide replacement reactions (Scheme 2). Upon addition of LiPh and Li[CCPh] to a solution of **2** at room temperature in non-coordinating solvents (hexane, pentane, benzene or toluene), the reaction mixture afforded orange crystals **5a** and **5b** in moderate yields after cooling at –30 °C overnight. The ^11^B NMR spectrum of **5a** showed a broad singlet at 121 ppm, slightly downfield-shifted from that observed for **2** (108 ppm). The chemical shift of the ^11^B NMR signal of **5b** is not significantly different from that of its precursor, lying at 107 ppm. Both signals of **5a** and **5b** fall within the range expected for transition metal boryl complexes,^12^,^16^,^27^,^28^ consistent with the expected products of the intended nucleophilic displacement reactions.

Single-crystal X-ray diffraction studies have confirmed the structures of **5a** and **5b** to be [(η^5^-C_5_H_5_)(OC)_2_Mn{μ-B(R)(*t*Bu)}Au(PPh_3_)] (R = Ph, **5a** and CCPh, **5b**), which are isostructural to **2**, apart from the second anionic substituent at boron.^13^ The Mn–B bond distances of **5a** and **5b** are 2.156(3) and 2.157(9) Å, respectively, very similar to each other and both slightly longer than that observed in **2** (2.126(9) Å). Concurrently, the Au–B bond also elongates slightly upon halide replacement, exhibiting Au–B distances of 2.356(2) and 2.326(8) Å for **5a** and **5b**, respectively, compared to 2.303(9) Å of **2**. The Mn–Au distances (**5a**: 2.548(1) Å; **5b**: 2.547(2) Å), on the other hand, are barely altered by halide substitution (*cf.* 2.553(1) Å). As observed in **2**, the boron centers of **5a** and **5b** adopt an almost planar geometry when the gold atom is disregarded (**5a**: ΣBα = 358.2°; **5b**: ΣBα = 358.6°).

**Fig. 1:**
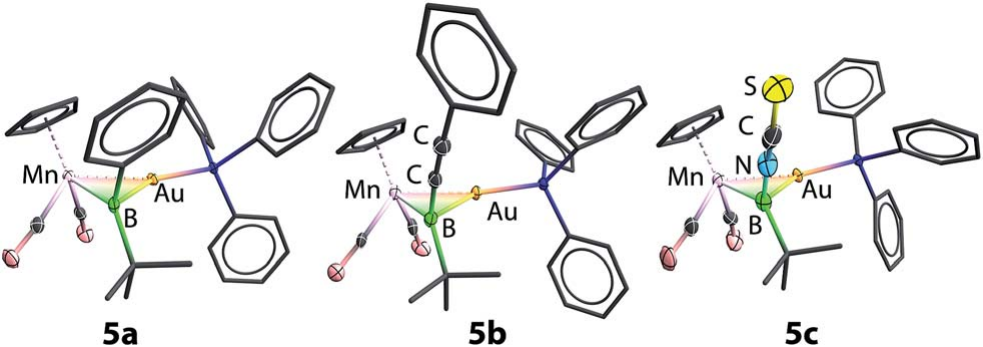
Molecular structures of **5a–c** in the solid state. Thermal ellipsoids are shown at 50% probability. H atoms and the ellipsoids of phenyl groups are omitted for clarity. Selected bond distances (Å) and angles (°) for **5a**: Mn–B 2.156(3), Mn–Au 2.548(1), Au–B 2.356(2), Au–P 2.293(1); P–Au–B 147.5(1), P–Au–Mn 160.2(1). For **5b**: Mn–B 2.157(9), Mn–Au 2.547(2), Au–B 2.326(8), Au–P 2.280(2); P–Au–B 140.4(2), P–Au–Mn 167.0(1). For **5c**: Mn–B 2.113(5), Mn–Au 2.555(1), Au–B 2.320(5), Au–P 2.291(1), B–N 1.532(7); P–Au–B 141.9(2), P–Au–Mn 166.1(1).

Similar reactions of **2** with [NBu_4_][NCS] led to a less selective reaction, from which a small amount of pale orange crystals (**5c**) were isolated from a complex mixture. Single-crystal X-ray diffraction studies confirmed the structure of this compound to be [(η^5^-C_5_H_5_)(OC)_2_Mn{μ-B(NCS)(*t*Bu)}Au(PPh_3_)] (**5c**), isostructural to **5a** and **5b** with an unusual boron-bound isothiocyanate substituent (Fig. 1).^29^ The Mn–B and Au–B distances of **5c** are 2.113(5) Å and 2.320(5) Å, noticeably shorter than those observed in **5a** and **5b**. The B–N distance of 1.532(7) Å falls between those established for aminoboranes R_2_B=NR_2_ and amine-borane adducts R_3_B–NR_3_, indicating significant π-donation from the nitrogen to boron. This is also reflected by the linear geometry (B–N–C 177.0(5)° and N–C–S 178.3(5)°) of the BNCS moiety. To the best of our knowledge, complex **5c** represents the first structurally characterized example of a transition metal complex bearing an isothiocyanate–substituted boryl ligand. The ^11^B NMR spectrum of **5c** showed a more upfield-shifted singlet at 95 ppm, which also falls within the range expected for transition metal boryl complexes.^26^,^30^

**Scheme 3:**
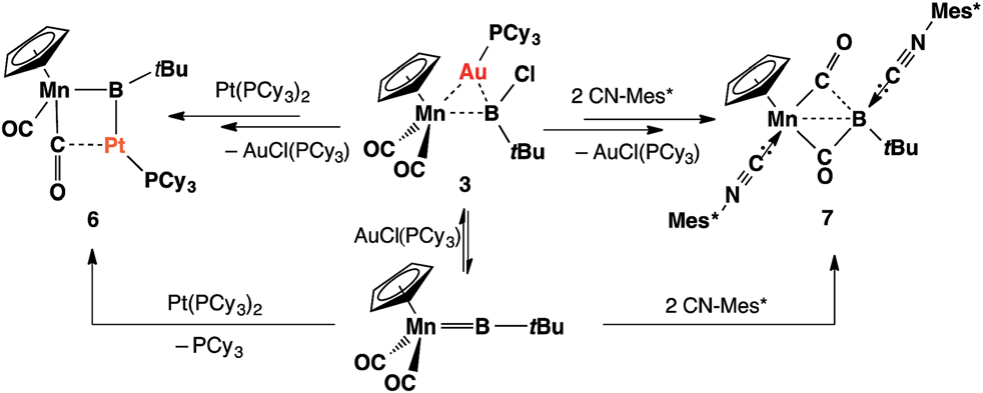
Formation of **6** and **7** from the reactions of **3** with [Pt(PCy_3_)_2_] and CNMes* (Mes* = 2,4,6-tri-*tert*-butylphenyl).

**Scheme 4:**
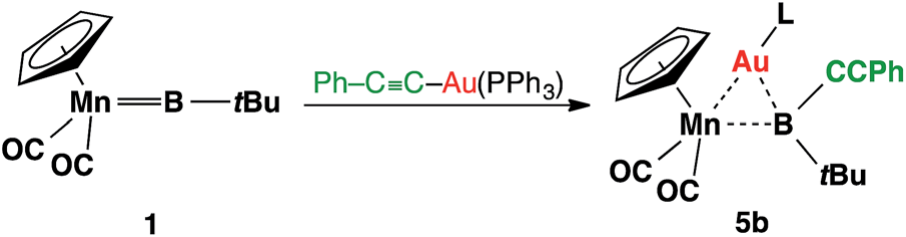
Transition-metal-mediated B–C coupling between gold acetylide and manganese borylene complexes.

Complex **3** shows similar reactivity towards nucleophiles. However, the expected products [(η^5^-C_5_H_5_)(OC)_2_Mn{μ-B(R)(*t*Bu)}Au(PCy_3_)] (R = Ph, CCPh and NCS) of the analogous reactions were in all cases found to be in equilibrium with the parent borylene complex **1** and [AuR(PCy_3_)] in the reaction mixtures, and thus could not be isolated. This liability of the [AuR(PCy_3_)] moiety in the [(η^5^-C_5_H_5_)(OC)_2_Mn{μ-B(Cl)(*t*Bu)}–Au(PCy_3_)] system was confirmed from the reaction of **3** with the platinum (0) complex [Pt(PCy_3_)_2_] and the isocyanide ligand CNMes* (Mes* = 2,4,6-tri-*tert*-butylphenyl, Scheme 3). In both reactions, known products [(η^5^-C_5_H_5_)(OC)_2_Mn{μ-CO}{μ-B(*t*Bu)}Pt(PCy_3_)] (**6**)^31^ and [(η^5^-C_5_H_5_)(Mes*NC)(OC)Mn{(CO)B(*t*Bu)(CNMes*)}] (**7**)^32^ were formed, in addition to [AuCl(PCy_3_)] (Scheme 3). These observations prompted the investigation of the direct reaction between **1** and the gold acetylide complex [Au(CCPh)(PPh_3_)], which afforded the acetylide–borylene coupling product **5b** in 61% yield (Scheme 4). Gold acetylides are well known to undergo C–C coupling reactions,^33^ and now have been shown to facilitate B–C bond formation, which presents a rare example of such reactivity.^34^

Complex **2** was also probed with sodium tetraarylborates for halide abstraction reactions. Upon addition of Na[BArCl4] to a toluene solution of **2**, with shaking, a cloudy orange solution was formed. After filtration and cooling at –30 °C over a week, the reaction mixture afforded orange crystals, which are insoluble in hexane, benzene or toluene, and moderately soluble in dichloromethane. The ^11^B NMR spectrum obtained from a CD_2_Cl_2_ solution showed a broad signal at 144 ppm and a sharp singlet at –7.04 ppm. The former is identical to that of the free borylene complex **1** (144 ppm), and the latter is attributed to the boron nuclei of the tetraarylborate counterion.

**Fig. 2:**
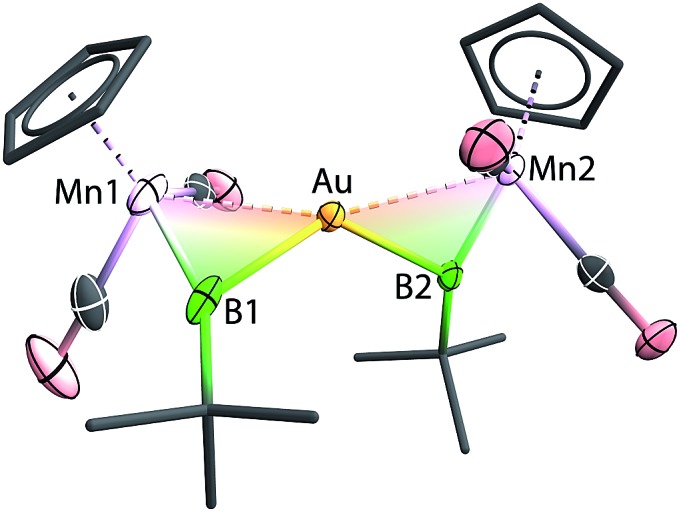
Molecular structure of [**8**]^+^ in the solid state. Thermal ellipsoids are shown at 50% probability. The counterion and the thermal ellipsoids of the Cp (Cp = η^5^-C_5_H_5_) and *t*Bu groups, as well as hydrogen atoms, are omitted for clarity. Selected bond distances (Å) and angles (°) for **8**: Mn1–B1 1.884(8), Mn1–Au 2.619(1), Au–B1 2.181(7), B1–C 1.555(7); Mn1–B1–C 156.4(5).

**Scheme 5:**
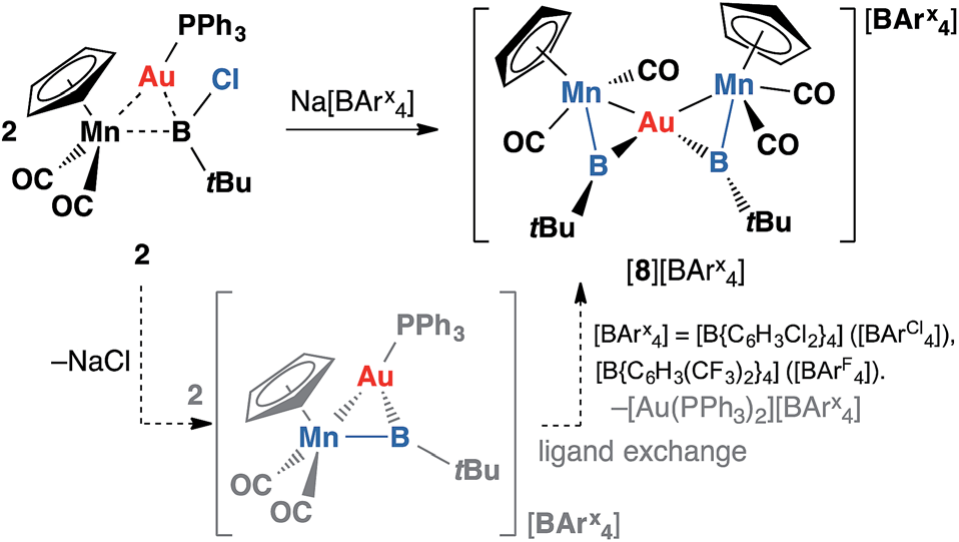
Formation of [{(η^5^-C_5_H_5_)(OC)_2_Mn}_2_{μ-B(*t*Bu)}_2_Au][BArx4] complexes [**8**][BArCl4] (Ar^Cl^ = C_6_H_3_Cl_2_) and [**8**][BArF4] (Ar^F^ = C_6_H_3_(CF_3_)_2_).

Instead of the expected product [{(η^5^-C_5_H_5_)(OC)_2_Mn}{μ-B(*t*Bu)}Au(PPh_3_)][BArCl4] (Ar^Cl^ = C_6_H_3_Cl_2_), single-crystal X-ray diffraction studies revealed the structure of these orange crystals to be [{(η^5^-C_5_H_5_)(OC)_2_Mn}_2_{μ-B(*t*Bu)}_2_Au][BArCl4] ([**8**][BArCl4]), comprising two borylene moieties coordinated to a single gold(i) cation (Fig. 2 and Scheme 5). The Mn–B distance of 1.884(8) Å is much shorter than those observed for the boryl complexes **2–5** (2.110–2.157 Å), and only marginally longer than the parent borylene complex **1** (1.810(9) Å). This suggests that the Mn–B interaction of [**8**]^+^ retains most of its multiple bond character while coordinated to the gold(i) cation, as also suggested by its ^11^B NMR signal. The Au–Mn distance of 2.619(1) Å, is noticeably longer than those observed for **5a–c** (2.548–2.555 Å), however, shorter than that observed in the neutral trimetallic gold boride complex [{(η^5^-C_5_H_5_)(OC)_2_Mn}_2_{μ-B(Au(PPh_3_))}] (2.651(4) Å), in which the Au(PPh_3_) moiety migrates between the two Mn–B parts rapidly even at –90 °C.^14^ The Au–B distance of 2.181(7) Å is considerably shorter than those of **5a–c** (2.320–2.356 Å). The angle between the two Mn–B–Au planes is 72.8°.

Initially, the formation of [**8**][BArCl4] was somewhat puzzling. Therefore, the reaction was repeated with an alternative halide abstracting agent Na[BArF4] (Ar^F^ = C_6_H_3_(CF_3_)_2_). From this reaction, besides the analogous product [{(η^5^-C_5_H_5_)(OC)_2_Mn}_2_{μ-B(*t*Bu)}_2_Au][BArF4] ([**8**][BArF4], Ar^F^ = C_6_H_3_(CF_3_)_2_), crystals of another product were also obtained, which was found to be [Au(PPh_3_)_2_][BArF4] (**12**) by X-ray crystallography. From this we proposed that the formation of [**8**][BArx4] (Ar^x^ = Ar^Cl^ and Ar^F^) proceeds *via* the expected cationic halide abstraction intermediate, followed by a ligand exchange, a route that has been reported for the analogous alkylidyne complexes (see Chart 1).^7^,^35^

**Scheme 6:**
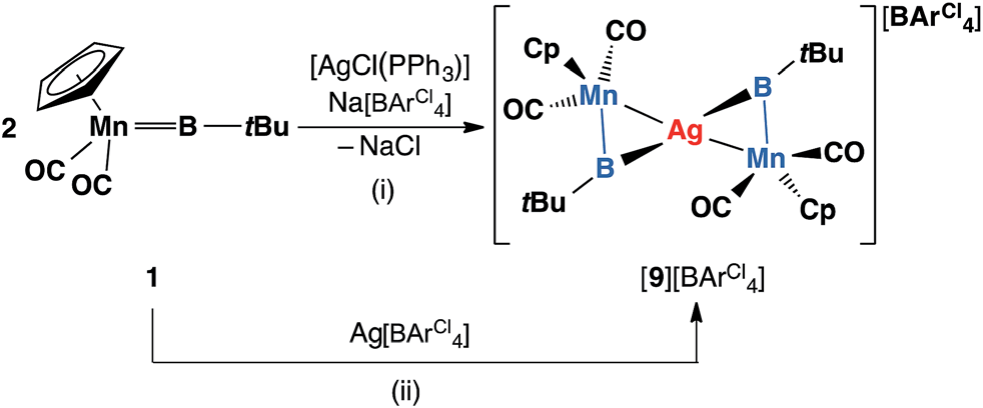
Syntheses of [**9**][BArCl4] *via* (i) salt elimination, and (ii) simple coordination (Cp = η^5^-C_5_H_5_).

Similar reactions have also been carried out with silver and copper salts. The reaction of **1** with [AgCl(PPh_3_)] in toluene led to formation of an inseparable mixture. However, in the presence of a stoichiometric amount of the halide abstracting agent Na[BArCl4], a mixture of 2 equiv. of **1** with [AgCl(PPh_3_)] led to formation of a mixture of colorless and orange crystals after being stored at –30 °C for 3–4 days. These crystals are extremely light-sensitive and thermally-unstable. Even in the strict argon atmosphere of a glovebox, black colloidal silver was observed inside and on the surface of crystals after being stored at room temperature. X-ray crystallographic studies revealed that this crystal mixture contained both the intended silver(i) product [{(η^5^-C_5_H_5_)(OC)_2_Mn}_2_{μ-B(*t*Bu)}_2_Ag][BArCl4] ([**9**][BArCl4]) and the by-product [Ag(PPh_3_)_3_][BArCl4] (Scheme 6(i)).

Complex [**9**][BArCl4] is sparingly soluble in toluene and moderately soluble in dichloromethane. It decomposes rapidly in solution, which precluded its purification. Consequently, we sought an alternative synthetic route with a view to a cleaner isolation of [**9**][BArCl4] in better yields. Direct coordination of the borylene complex **1** to Ag(i) was found to be possible in the presence of a non-coordinating counterion. The reaction of **1** and Ag[BArCl4] led to clean formation of [**9**][BArCl4] in 90% yield (Scheme 6(ii)). The NMR spectra of this compound are almost identical to those of [**8**][BArCl4] for all observable nuclei.

**Fig. 3:**
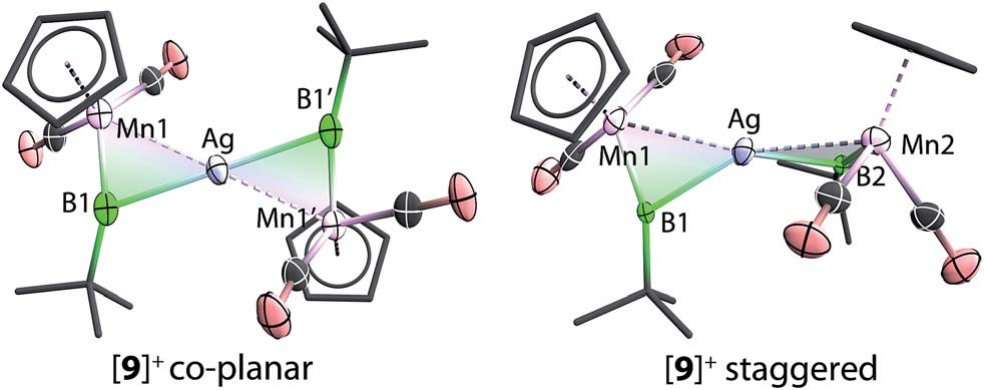
Molecular structures of [**9**]^+^ in the solid state. Thermal ellipsoids are shown at 50% probability. The counterions and the thermal ellipsoids of Cp and *t*Bu groups, as well as hydrogen atoms, are omitted for clarity. Selected bond distances (Å) and angles (°) for [**9**]^+^ coplanar: Mn1–B1 1.843(6), Mn1–Ag 2.688(1), Ag–B1 2.456(6), B1–C 1.555(7), Mn1–B1–C 162.8(5). For [**9**]^+^ staggered: Mn1–B1 1.856(4), Mn1–Ag 2.680(7), Ag–B1 2.289(12), B1–C 1.519(4), Mn1–B1–C 1.856(4), angle between planes Ag–B1–Mn1 and Ag–B2–Mn2 67.8(8).

The solid-state structures of [**9**]^+^ are shown in Fig. 3. In comparison to [**8**]^+^, in which the two borylene moieties are staggered, the two three-membered ring moieties (the atoms Mn1, B1, Mn2, B2 and Ag) of [**9**]^+^ were found in both coplanar and staggered geometries from different crystal samples. This suggests that the energy difference between these two geometries is insignificant, which has been confirmed by computations (*vide infra*). In the staggered form, the angle between the two Ag–B–Mn planes is 67.8(8)°.

**Fig. 4:**
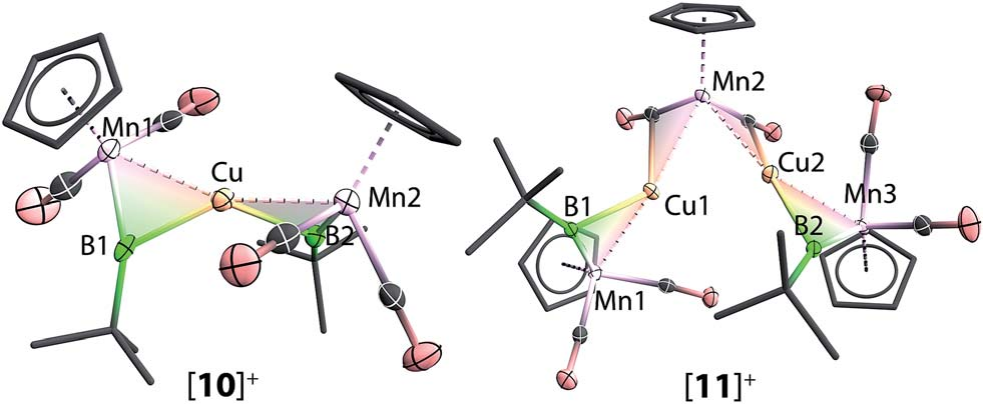
Molecular structures of [**10**]^+^ and [**11**]^+^ in the solid state. Thermal ellipsoids are shown at 50% probability. Counterions, the thermal ellipsoids of the Cp and *t*Bu groups, as well as hydrogen atoms, are omitted for clarity. Selected bond distances (Å) and angles (°) for [**10**]^+^: Mn1–B1 1.850(6), Mn1–Cu1 2.447(1), Cu1–B1 2.146(5), B1–C 1.549(7), Mn2–B2 1.857(5), Mn2–Cu1 2.454(1), Cu1–B2 2.178(5), B2–C 1.541(6), Mn1–B1–C 162.1(3), Mn2–B2–C 161.0(3), angle between planes Cu–B1–Mn1 and Cu–B2–Mn2 65.4(3). For [**11**]^+^: Mn1–B1 1.865(4), Mn1–Cu1 2.473(1), Cu1–B1 2.123(3), B1–C 1.516(4), Mn2–B2 1.865(3), Mn2–Cu2 2.445(1), Cu2–B2 2.128(3), B2–C 1.549(4), Mn3–Cu1 2.5184(9), Mn3–Cu2 2.497(1), Cu1–Cu2 2.883(1), Cu1–C1 2.282(3), Cu2–C2 2.356(3); Mn1–B1–C 159.8(2), Mn2–B2–C 160.6(2).

**Scheme 7:**
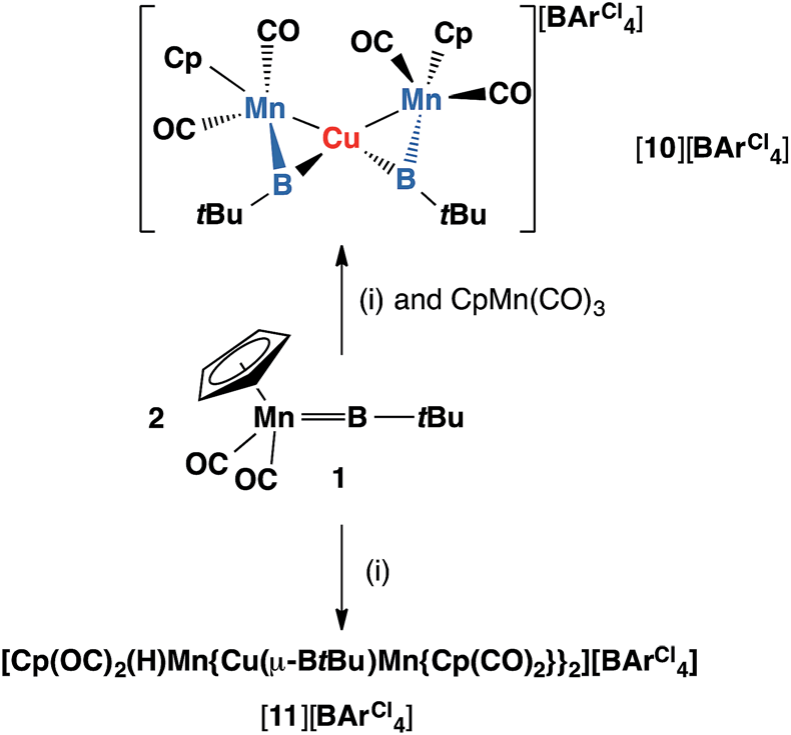
Formation of copper complexes [**10**][BArCl4] and [**11**][BArCl4] from reactions of **1** and (i), where (i) = [CuCl(PPh_3_)], Na[BArCl_4_].

Reactions of **1** with [CuCl(PPh_3_)] and sodium tetraarylborate Na[BArCl4] in toluene or fluorobenzene were less selective compared to those involving silver(i) complexes. In both solvents, small yields of crystals were isolated. X-ray crystallography revealed the solid state structure of these crystals to be [**11**][BArCl4], where [**11**]^+^ consists of two metal–borylene-coordinated copper moieties connected by a [MnH(η^5^-C_5_H_5_)(CO)_2_] fragment, which makes it a diamagnetic compound (Fig. 4 and Scheme 7). Due to the presence of metal atoms, it was not possible to locate the position of the hydride unambiguously (See ESI†). In attempts to increase the selectivity of the reaction to favour the formation of [**11**][BArCl4], one equivalent of cymantrene [Mn(η^5^-C_5_H_5_)(CO)_3_] was added to the reaction of **1** with [CuCl(PPh_3_)] and Na[BArCl_4_] in toluene. This reaction afforded low yields of orange crystals, which was confirmed to be the originally-intended product [{(η^5^-C_5_H_5_)(OC)_2_Mn}_2_{μ-B(*t*Bu)}_2_Cu][BArCl4] ([**10**][BArCl4]) by X-ray crystallography. Crystals of **10** and **11** are sparingly soluble in benzene and toluene. Although moderately soluble in dichloromethane, the solutions decompose within a minute at room temperature. Due to this extreme instability and low yields of the products, no useful NMR data could be obtained. Subsequently both [**10**][BArCl4] and [**11**][BArCl4] could only be characterized by X-ray crystallography (see ESI†).

The important bond distances angles of the cations [**8**]^+^, [**9**]^+^, [**10**]^+^ and [**11**]^+^ are summarized in Table 1. Similar to [**8**]^+^ mentioned earlier, the Mn–B distances in [**9**]^+^ (1.851(7) Å), [**10**]^+^ (1.850(6) Å and 1.857(5) Å) and [**11**]^+^ (1.865(4) Å) are only slightly longer than that of the terminal borylene complex **1** (1.810(9) Å) and much shorter than a Mn–B single bond.^36^,^37^ Furthermore, the Mn–B–C angles of [**8**]^+^–[**11**]^+^ range from 156.4° to 162.7° (*cf.* in **1**: 174.3°). These structural features are in contrast to all other homo- and hetero-dinuclear bridging borylenes containing the same [MnBR] fragment, in which the Mn–B distances are considerably longer (1.91–2.02 Å) and the Mn–B–C angles are significantly more acute (135.2–145.8°).^37^,^38^ These data suggest a strong borylene character of the [(η^5^-C_5_H_5_)(OC)_2_Mn(μ-B*t*Bu)] moiety, (*i.e.* the borylene moiety is more semi-bridging), which is consistent with the ^11^B NMR data. Furthermore, The Mn–M distances of [**9**]^+^ (M = Ag) and [**10**]^+^ (M = Cu) are 2.683(1) Å and 2.447(1) Å respectively (*cf.* Mn–Au 2.619(1) Å in [**8**]^+^), consistent with the trend of the atomic radii of Au, Ag and Cu.^39^,^40^ The dihedral angles of [**8**]^+^, [**9**]^+^, and [**10**]^+^ are listed in Table 1. These are in a comparable range to those of the isoelectronic alkylidyne analogue [W_2_Au(μ-CC_6_H_4_Me-4)_2_(CO)_4_(η-C_5_H_5_)_2_]^+^] (62°).^41^

**Table 1:** Selected bond distances (Å) and angles (°) of complexes **8**, **9**, **10** and **11** in the solid state (M = Au, Ag, Cu)

	[**8**]^+^^*a*^ (Mn_2_B_2_Au)	[**9**]^+^^*b*^ (Mn_2_B_2_Ag)	[**10**]^+^ (Mn_2_B_2_Cu)	[**11**]^+^ (Mn_3_Cu_2_B_2_)
Mn1–B1	1.884(8)	1.851(7)	1.850(6)	1.865(4)
Mn2–B2	—	—	1.857(5)	1.865(3)
Mn1–M	2.619(1)	2.683(1)	2.447(1)	2.473(1)
Mn2–M	—	—	2.454(1)	2.445(1)
M–B1	2.181(7)	2.456(6)	2.178(5)	2.123(3)
M–B2	—	—	2.146(5)	2.128(3)
Mn1–B1–C	156.4(6)	162.7(5)	162.1(4)	160.6(2)
Mn2–B2–C	—	—	161.0(4)	159.8(2)
Mn1–B1–B2–M2	72.8(4)	67.8(8)	65.4(3)	—

^*a*^Due to disorder of the molecule in the solid state, the bond distances and angles were taken from the less affected half of the molecule.

^*b*^Only half of the cation **9** is present in the asymmetric unit. The other part is generated by inversion symmetry.

To gain more insight into the bonding of complexes **8–10**, DFT computations were also performed.^42^ The energy minima found for all three cations [**8**]^+^–[**10**]^+^ corresponded to the observed non-planar geometry of the M(MnB)_2_ cores. The optimization and subsequent harmonic frequency calculations of the coplanar *C*_*i*_ structure observed for [**9**]^+^ showed it to be a transition state on the energy hypersurface. The energy difference between the co-planar and staggered form is very small (3 kcal mol^–1^). This is presumably due to the closed-shell d^10^ centre of Ag^+^, the geometry of which is not dictated by crystal field effects.

No localized two-center, two-electron bonds (2c, 2e bonds) between the central metal and the borylene ligands have been found using the Natural Bond Orbital (NBO) method. However, strong donor–acceptor interactions have been found using the second-order perturbation analysis. The dominating interactions are the donations from the Mn–B bonds to a low-populated coinage-metal-centred orbital. The backdonation to the borylene from the coinage metals is an order of magnitude weaker. Computations carried out employing gross populations of fragment orbitals have shown that charge donation from the borylene ligands to the coinage metals decreases from Cu to Au (1.90, 1.60 and 1.51 e), whereas the backdonation grows from Cu to Au (0.56, 0.57 and 1.12 e respectively). The first of these effects can be ascribed to the increasing size of the *n*s orbital of coinage metal, which leads to its smaller overlap with the π-type orbital of the borylene unit. For the backbonding, the coinage metal delivers electrons from its (*n* – 1)d orbital to the empty π* orbital of borylene. In the case of copper the 3s,p and 3d orbitals have similar size, thus the 3d → π* interaction is weakened by repulsion with the 3s,p orbitals, which have symmetries incompatible with this interaction. When changing to Ag, the orthogonality of the 4d and 3d orbitals causes an increase of the radii of the 4d orbitals. The spatial separation of the 4s,p and the 4d orbitals is larger and thus the repulsion is smaller, while the orbital overlap is better. In the case of Au, the relativistic contraction additionally amplifies this effect and therefore results in the strongest backdonation.^43^

The weak backdonation from the coinage metal cations to borylene may be responsible for its semibridging geometry and preserved strong borylene character (short Mn=B bond and large Mn–B–R angle). In turn, the semibridging geometry and the slight preference of the staggered geometry may play a role in maximising backdonation from the coinage metal cations to the borylene in a similar way to the bisphosphine gold(i) system [(DPCb)AuCO]^+^ (DPCb = *o*-carborane diphosphines) reported by Amgoune, Bourissou and co-workers. It has been observed that ligands in Au(i) complexes that deviate from linear geometry cause the energies of the 5d orbitals to rise, which strengthens the backdonation from the Au(i) center to the ligand (CO).^44^ It is interesting to note that the structural differences observed between borylene coordination to coinage metals and other transition metal fragments closely reflect those reported for analogous alkylidyne chemistry: when coordinated to Au^+^, alkylidynes also exhibit larger M–_μ_C–R angles and shorter M–_μ_C bonds than bridging alkylidynes with most other metals, where the _μ_CR vector is closer to perpendicular to the M–Au bond^4^,^45^–^50^ These findings further demonstrate the unique bonding characteristics of coinage metals.

**Fig. 5:**
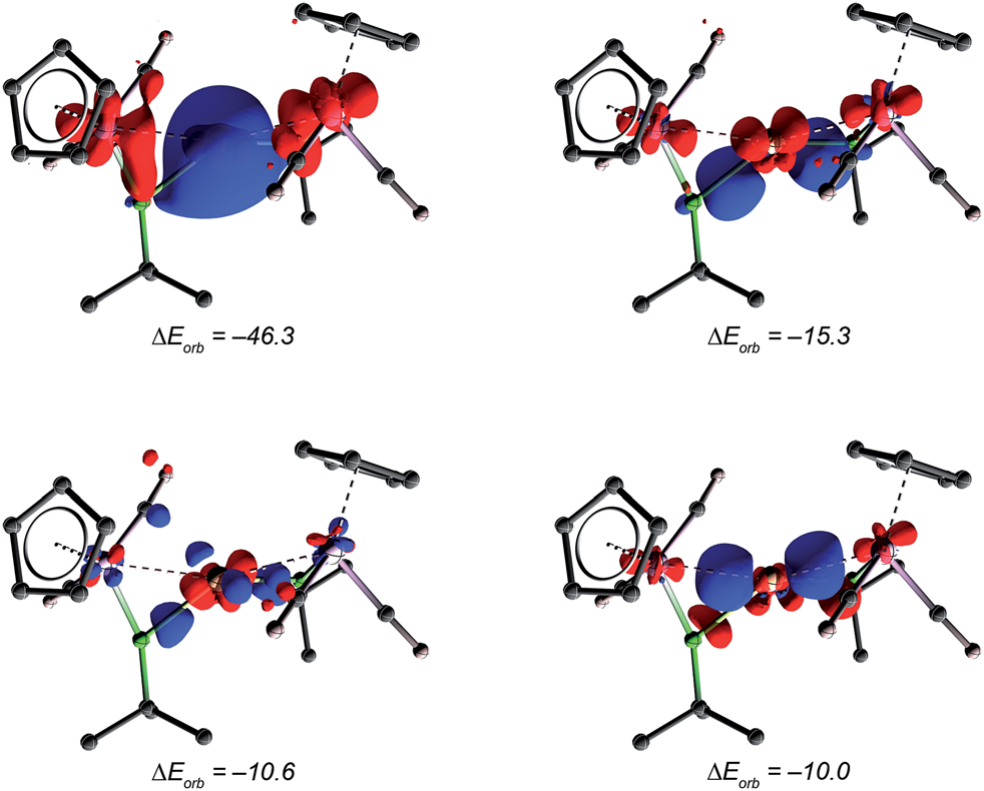
Contours of deformation density representing the borylene–copper interaction in [**10**]^+^ with the corresponding orbital interactions energies [kcal mol^–1^]. The most significant describes borylene-to-Cu π-donation. The other three weaker interactions account mainly for the backdonation from the copper center to boron, carbon and manganese atoms respectively. The *iso*-value used for this graphic was set to 0.002 a.u. The red coloration indicates electron density depletion, blue concentration.

Further bonding analysis has been carried out by employing the ETS-NOCV decomposition scheme as implemented in the ADF software package. The four most significant deformation densities of [**10**]^+^ are depicted in Fig. 5 (see ESI† for figures of orbitals of the fragments). The strongest interaction of –46.3 kcal mol^–1^ originates from donation of the borylene HOMO and HOMO–2 orbitals into the LUMO of copper (π-Mn=B → σ-Cu^+^) and the minor backdonation from the copper HOMO into the LUMO of the borylene moieties (*d*_*xz*_ Cu^+^ → π*-Mn=B). This is in contrast to the semi-bridging aminoborylene complexes [L_n_M{μ-BN(SiMe_3_)_2_}M′(CO)_5_] (L_n_M = (η^5^-C_5_Me_5_)(OC)Ir, M′ = Cr, W) and [(OC)_3_(L)Mo{μ-BN(SiMe_3_)_2_}{μ-CO}M′(PCy_3_)} (L = CO, PMe_3_, M′ = Pd, Pt), in which the M–B π-interactions (M = Ir and Mo) are heavily compromised due to the strong B=N π-interaction. As such, in aminoborylene-bridged complexes, the second metal centre (Cr/W and Pd/Pt, respectively) interacts predominantly with the boron atom instead of the metal–borylene ligand as a whole.^23^

**Fig. 6:**
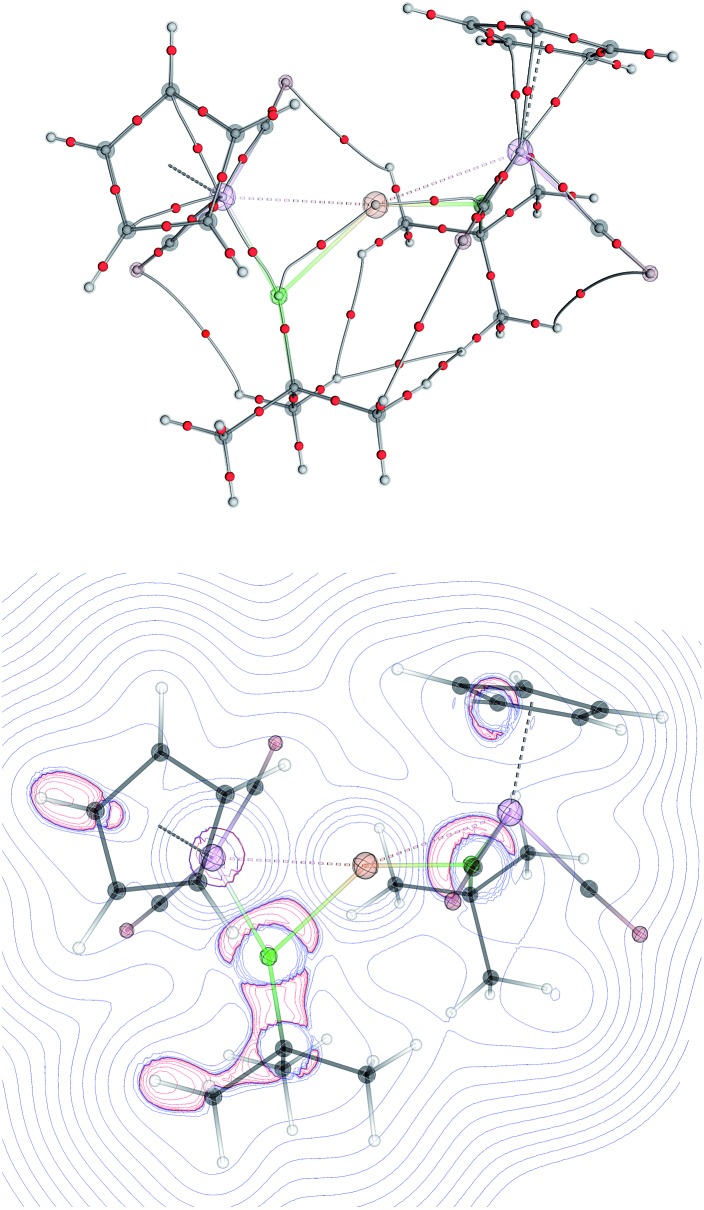
Topology of ∇^2^*ρ* of the cation [**10**]^+^ obtained from atoms-in-molecules analysis. (Top) Bond paths with bond critical points are represented as red dots and the atomic attractors as grey dots. (Bottom) Cross-cut of ∇^2^*ρ* in the [MnBCu] plane showing valence shell charge concentration (VSCC) near a boron atom. The geometry of cation [**10**]^+^ is overlaid as transparency.

The topology of the Laplacian of electron density (∇^2^*ρ*) was analyzed by means of Bader's theory of Atoms in Molecules (AiM) for [**8**]^+^–[**10**]^+^. Despite the bonding interactions between the Mn and M (M = Au, Ag and Cu) evident in the structural data, no bond critical points (BCP) between the manganese and the coinage metal cations, nor any ring critical points, could be found for the three-membered M{MnB} rings, similar to previously reported multi- and dinuclear borylene-bridged complexes.^14^,^23^ The only localized BCPs were those for the Mn–B and M–B (M = Cu, Ag, Au) bonds. Similarly to the previously-reported gold boryl complex **2**,^13^,^14^ the bond paths from the boron to the coinage metal cations in [**8**]^+^–[**10**]^+^ are strongly bent towards the manganese atom, suggesting that the interactions between the boron and coinage-metal atoms are not covalent but rather σ-donation from π-orbitals of the manganese borylene unit. The valence shell charge concentration (VSCC) close to the boron atom is extended over both metal–boron BCPs (Mn–B and Au–B; see Fig. 6). These findings and the calculated Wiberg Bond Indices (Table 2) support the bonding picture of side-on-coordinated manganese borylenes to coinage metal cations. The interactions between the metal atoms are more electrostatically-dominated while those between the boron atoms and coinage metal cations are somewhat more covalent in character.

**Table 2:** Natural charges and Wiberg Bond Indices of cations [**8**]^+^–[**10**]^+^ determined by NBO analysis

	[**8**]^+^ (M = Au)	[**9**]^+^ (M = Ag)	[**10**]^+^ (M = Cu)
**Natural charge**			
M	0.50	0.67	0.69
Mn	–0.56	–0.60	–0.71
B	0.66	0.74	0.73

**Wiberg bond indices (WBI)**			
M–Mn	0.14	0.10	0.10
M–B	0.42	0.25	0.25
Mn–B	0.77	0.88	0.85

## Conclusions

In conclusion, we have demonstrated that both metal boryl and borylene complexes can serve as side-on coordinated “metalloligands” to the coinage metals to form isolobal analogs of classical π complexes and Stone's alkylidene- and alkylidyne-bridged multinuclear complexes. The first structural examples of bis(metal–borylene) coordinated coinage metal complexes have been presented and their bonding has been thoroughly examined using DFT calculations, in which the terminal metal–borylene moieties are treated as a ligand as a whole for the first time.

## Supplementary Material

Supplementary information

Crystal structure data
