# Supplementary material for: Side-on coordination of boryl and borylene complexes to cationic coinage metal fragments
Source: Chem Sci. 2015 Mar 10;6(5):2989–96. doi: 10.1039/c5sc00211g (PMC5729409; doi:10.1039/c5sc00211g)
Supplement: Supplementary file 1 [file SC-006-C5SC00211G-s001.pdf]

Electronic Supplementary Material (ESI) for Chemical Science.
This journal is © The Royal Society of Chemistry 2015

Supporting Information

Side-on Coordination of Boryl and Borylene Complexes to Cationic Coinage Metal Fragments

Holger Braunschweig*, Krzysztof Radacki, and Rong Shang

Institut für Anorganische Chemie

Julius-Maximilians-Universität Würzburg

Am Hubland, 97074 Würzburg (Germany)

Fax: (+49)931-31-84623

E-Mail: h.braunschweig@uni-wuerzburg.de

Table of Contents

Experimental Section 2

Crystal structure determination 6

Computational Studies 11

General considerations 11

Natural Bond Orbital Analysis (NBO) for cation $[10]^+$ 12

Electron Localization Function (ELF) Topology for cation $[10]^+$ 14

Energy Decomposition Analysis (EDA) of $[8]^+$–$[10]^+$ 15

Charges $[8]^+$–$[10]^+$ 16

Atoms in Molecules (AiM) – Topology of $\nabla^2\rho$ for cation $[8]^+$–$[10]^+$ 17

ETS-NOCV analysis of $[10]^+$ 19

Comparison of ETS-NOCV results for $[8]^+$–$[10]^+$ 21

Calculated and experimental IR-spectra of $[11]^+$ 23

References 25

Experimental Section

General considerations

All syntheses were carried out under an argon atmosphere with standard Schlenk and glovebox techniques unless otherwise stated. The complex [(η^5-C_5H_5)(OC)$_2$Mn=B(*t*Bu)] (**1**) and the gold(I) complexes were prepared according to published procedures.[1-3] Pentane, hexane and benzene were dried by distillation over Na/K alloy under argon and stored over molecular sieves. Toluene and C_6D_6 were dried over Na, distilled under argon and stored over molecular sieves. Elemental analyses were obtained from an Elementar Vario MICRO cube instrument. NMR spectra were recorded on a Bruker Avance 400 (^{1}H: 400 MHz, ^{31}P{^{1}H}: 162 MHz, ^{11}B: 128 MHz, ^{13}C{^{1}H}: 101 MHz) and/or a Bruker Avance 500 FT-NMR spectrometer (^{1}H: 500 MHz, ^{31}P{^{1}H}: 202 MHz, ^{11}B: 160 MHz, ^{13}C{^{1}H}: 126 MHz). Chemical shifts are given in ppm, and are referenced against external $SiMe_4$ (^{1}H, ^{13}C{^{1}H}), $BF_3{\cdot}Et_2O$ (^{11}B) and 85% H_3PO_4 (^{31}P).

Preparation of [(η^5-C_5H_5)(OC)$_2$Mn{μ-B(R)(*t*Bu)}Au(PPh$_3$)] (R = Ph, C≡CPh and NCS)

In a glovebox charged with an argon atmosphere, a solid mixture of the gold-manganese complex [(η^5-C_5H_5)(OC)$_2$Mn{μ-B(Cl)(*t*Bu)}Au(PPh$_3$)] (**2**) (5 mg, 0.007 mmol) and a stoichiometric amount of a nucleophile (PhLi, 0.7 mg, 0.007 mmol; [NBu$_4$][SCN], 0.8 mg, 0.007 mmol or LiCCPh, 0.8 mg, 0.007 mmol) was dissolved in *ca*. 1.5 mL of benzene. The reaction mixture was shaken by hand for three minutes and the resulting intense orange solution was filtered immediately through a cotton plug. The bright orange filtrate was collected in a small vial, to which *ca.* 1.5 mL of pentane was added. The solution mixture was stored at –30 °C overnight. As the benzene slowly froze from the solvent mixture, X-ray quality crystals of [(η^5-C_5H_5)(OC)$_2$Mn{μ-B(R)(*t*Bu)}Au(PPh$_3$)] (R = Ph, CCPh and NCS) formed in varying yields.

[(η^5-C_5H_5)(OC)$_2$Mn{μ-B(Ph)(*t*Bu)}Au(PPh$_3$)] (5a): 4 mg, 78% yield. IR (solid): 1913 (s), 1851 (s) (νCO) cm^{-1}. ^{1}H NMR (C_6D_6): δ_H 7.50–6.98 (m, 20 H, Ph) 4.38 (s, 5 H, η^5-C_5H_5), 1.63 [s, 9 H, BC(CH_3)$_3$]. ^{13}C{^{1}H} NMR (C_6D_6): δ_C 234.2 (s, br, *C*O), 225.5 (s, br, *C*O) 134.21 (d, J_{PC} = 14, PPh), 131.41 (d, J_{PC} = 2, PPh), 131.10 (d, J_{PC} = 45.3, PPh), 129.42 (d, J_{PC} = 10, PPh), 129.03 (br, BPh), 126.26 (s, BPh), 123.01 (s, BPh), 86.84 (η^5-C_5H_5), 37.54 (br, B*C*(CH$_3$)$_3$), 30.82 (s BC(*C*H$_3$)$_3$). ^{11}B NMR (C_6D_6): δ_B 120.3 (s, br). ^{31}P{^{1}H} NMR (C_6D_6): δ_P 55.0 (s). Anal. found: C, 54.24; H, 4.26. Calcd. for $C_{35}H_{34}O_2AuMnPB$: C, 53.87; H, 4.39.

[(η^5-C_5H_5)(OC)$_2$Mn{μ-B(C≡CPh)(*t*Bu)}Au(PPh$_3$)] (5b): 5 mg, 83% yield. IR (solid): 1913 (s), 1863 (s) (νCO) cm^{-1}. ^{1}H NMR (C_6D_6): δ_H 7.51–6.94 (m, 20 H, Ph), 4.82 (5 H, η^5-C_5H_5), 1.78 (9 H, BC(CH_3)$_3$); ^{1}H NMR (500 MHz, C_6D_6): δ_H 7.51–7.43, 7.13–6.92 (m, 20 H, Ph), 4.82 (s, η^5-C_5H_5), 1.78 (s, 9 H, BC(CH_3)$_3$); ^{13}C{^{1}H} NMR (125.8 MHz, C_6D_6): δ_C 233.5 (s, br, CO), 226.4 (s, br, CO), 134.4 (d, J_{PC} = 14, PPh), 131.6 (s, CPh), 131.3 (d, J_{PC} = 2, *p*-Ph), 130.8 (d, J_{PC} = 48, *ipso*-Ph), 129.4 (d, J_{PC} = 11, Ph), 126.6 (s, CPh), 125.7 (s, CPh), 106.7 (s, *C*Ph), 87.26 (η^5-C_5H_5), 37.26 (s, br, B*C*(CH$_3$)$_3$), 31.63 (s, BC(*C*H$_3$)$_3$); ^{11}B NMR (128 MHz, C_6D_6): δ_B 107.8 (s, br); ^{11}B NMR (161 MHz, C_6D_6): δ_B: 105.5 (s, br). ^{31}P{^{1}H} NMR (162 MHz C_6D_6): δ_P 53.4 (s) ^{31}P{^{1}H} NMR (202 MHz, C_6D_6): δ_P 53.4 (s). Anal. found: C, 58.44; H, 4.47. Calcd. for $C_{37}H_{34}O_2$MnAuPB·C_6H_6: C, 58.52; H, 4.57.

[(η^5-C_5H_5)(OC)$_2$Mn{μ-B(NCS)(*t*Bu)}Au(PPh$_3$)] (5c): Crystals form from the reaction mixture 20% of the time in around 20% yield (ca. 1 mg). IR (solid): 1921 (s), 1867 (s) (νCO) cm^{-1}. ^{1}H NMR (C_6D_6): δ_H 7.39–6.96 (m, 15 H, Ph), 4.63 (5 H, η^5-C_5H_5), 1.41 (BC(CH_3)$_3$), ^{13}C{^{1}H} NMR (125.8 MHz, C_6D_6): 134.6 (N*C*S), 134.3 (d, J_{PC} = 14, PPh), 131.6 (d, J_{PC} = 2, *p*-Ph), 130.4 (d, J_{PC} = 49, *ipso*-Ph), 129.6 (d, J_{PC} = 11, Ph), 85.56 (s, η^5-C_5H_5), 37.26 (s, br, B*C*(CH$_3$)$_3$), 29.59 (s, BC(*C*H$_3$)$_3$); ^{11}B NMR (C_6D_6): δ_B 95.1 (s, br). ^{31}P{^{1}H} NMR (C_6D_6): δ_P 53.6 (s).

Reactions of 3 with nucleophiles

The reactions of **3** with nucleophiles LiPh, LiCCPh and [NBu$_4$][NCS] have also been carried out in the same way as what has been described for **2**. However the reactions lead to mixtures of compounds from which only [(η^5-C_5H_5)(OC)$_2$Mn{μ-B(CCPh)(*t*Bu)}Au(PCy$_3$)] (**6**) could be partially characterized. ^{1}H NMR (C_6D_6): δ_H 7.53–7.00 (m, 6 H, Ph), 4.83 (5 H, η^5-C_5H_5), 1.87 (9 H, BC(CH_3)$_3$), 1.77–0.95 (33 H, Cy) ^{11}B NMR (C_6D_6): δ_B 106.4 (s, br). ^{31}P{^{1}H} NMR (C_6D_6): δ_P 70.9 (s).

Preparation of [{(η^5-C_5H_5)(OC)$_2$Mn}$_2${μ-B*t*Bu}$_2$Au][BAr$^x_4$], Ar^x = 3,5-$C_6H_3Cl_2$ ([8][BAr$^{Cl}_4$]) and 3,5-$C_6H_3(CF_3)_2$ ([8][BAr$^F_4$])

In a glovebox charged with an Ar atmosphere, a solid mixture of the gold borylene complex **2** (5 mg, 0.007 mmol) and a stoichiometric amount of Na[BAr$^x_4$] (Ar^x = $C_6H_4Cl_2$, 4 mg, 0.007 mmol; Ar^x = $C_6H_4(CF_3)_2$, 5 mg, 0.007 mmol) was dissolved in ca. 1.5 mL of toluene. The reaction mixture was shaken by hand for three minutes and the resulting intense orange solution was filtered immediately through a cotton plug. The bright orange filtrate was collected in a small vial, to which *ca.* 1.5 mL of *n*-pentane was added. The solution mixture

was stored at −30 °C for 5 days. X-ray quality crystals of [**8**][BAr^{Cl}_4] formed in satisfactory yield (4 mg, 40%), which are sparingly soluble in *n*-hexane, benzene, toluene, and soluble in DCM. When Na[$C_6H_4(CF_3)_2$] was used, the reaction mixtures afforded a mixture of crystals in low yields. An analytically pure sample of [**8**][BAr^F_4] could not be obtained.

[{(η^5-C_5H_5)$(OC)_2$Mn}$_2${μ-B*t*Bu}$_2$Au][BAr^{Cl}_4] ([8][BAr^{Cl}_4]): ^{1}H NMR (CD_2Cl_2): δ_H 7.04-7.03 (8 H, m, br, *o*-$C_6H_3Cl_2$), 7.00-7.04 (4 H, s, *p*-$C_6H_3Cl_2$), 5.17 [10 H, s, η^5-C_5H_5], 1.25 (18 H, s, $BC(CH_3)_3$); $^{13}C\{^1H\}$ NMR (CD_2Cl_2): δ_C 219.1 (s, CO), 133.5 (m, br, *o*-$C_6H_3Cl_2$), 133.3 (q, J_{BC} = 5, *m*-$C_6H_3Cl_2$), 123.4 (s, *p*-$C_6H_3Cl_2$), 85.98 (s, η^5-C_5H_5), 26.41 (s, $BC(CH_3)_3$); ^{11}B NMR (CD_2Cl_2): δ_B 144.3 (s, br), −7.04. Anal. Found: C, 43.65; H, 3.08. Calcd. for $C_{46}H_{40}AuMn_2Cl_8O_4B_3$: C, 43.17; H, 3.15.

Preparation of [{(η^5-C_5H_5)$(OC)_2$Mn}$_2${μ-B(*t*Bu)}$_2$Ag][B{$C_6H_3Cl_2$}$_4$] ([9][BAr^{Cl}_4])

Method 1: In a glovebox charged with an argon atmosphere, to a solid mixture of the borylene complex [(η^5-C_5H_5)$(OC)_2$Mn=B*t*Bu] (**1**, 10 mg, 0.041 mmol) and Ag[B$(C_6H_3Cl_2)_4$] (14 mg, 0.021 mmol) was added *ca.* 4 mL toluene. This slurry was shaken vigorously until a cloudy orange solution was obtained, which was then quickly filtered through a cotton plug. The clear orange filtrate was left at room temperature until it started to crystallize (*ca.* 1 min) before moving it to the freezer for further crystallization. Brown crystals of the title compound formed overnight from the solution in 90% yield (22 mg).

Method 2: In a glovebox charged with an argon atmosphere, to a solid mixture of the borylene complex [(η^5-C_5H_5)$(OC)_2$Mn=B*t*Bu] (**1**, 20 mg, 0.082 mmol), [AgCl(PPh_3)] (17 mg, 0.41 mmol) and Na[B$(C_6H_3Cl_2)_4$] (25 mg, 0.41 mmol) was added *ca.* 3 mL of fluorobenzene. The resulting cream-colored slurry was shaken by hand for 5 minutes resulting in a light orange cloudy solution, which was filtered immediately through a cotton plug. The clear orange filtrate was collected in a small vial and stored at –30 °C overnight, to which added toluene and pentane (0.5 mL each). This mixture was stored at –30 °C for another two days. A mixture of yellow and colorless crystals formed in the vial, which consisted of the title compound and [Ag$(PPh_3)_3$][B{$C_6H_3(CF_3)_2$}$_4$] (yield of the crystal mixture: 0.35 mg). Crystals were only obtained when toluene and pentane were added to the solution.

[(η^5-C_5H_5)$_2$$(OC)_4$$Mn_2$Ag(μ-B*t*Bu)$_2$][$BAr^{Cl}_4$] ([9][$BAr^{Cl}_4$]): ^{1}H NMR ($CD_2Cl_2$): δ_H 7.40-7.05 (m, 12 H, B$(C_6H_3Cl_2)_4$), 5.04 [10 H, η^5-C_5H_5], 1.21 [18 H, $BC(CH_3)_3$]; $^{13}C\{^1H\}$ NMR (CD_2Cl_2): δ_C 133.7, 133.4, 123.9 (br, B(*C*$_6H_3Cl_2$)$_4$), 84.83 (s, η^5-*C*$_5H_5$), 25.57 (s, BC(*C*H$_3$)$_3$). ^{11}B NMR

(CD_2Cl_2): δ_B 145.6 (Mn=B), −7.06 (BAr^{Cl}_4). Anal. Found: C, 48.72; H, 3.58. Calcd. for $C_{46}H_{40}AgMn_2Cl_8O_4B_3 \cdot C_6H_5F$: C, 48.54; H, 3.53.

[Ag(PPh$_3$)$_3$][BAr$^{Cl}_4$] ^1H NMR (CD_2Cl_2): δ_H 7.61-6.99 (m). ^{11}B NMR (CD_2Cl_2): δ_B −6.97 (BAr^{Cl}_4); $^{31}P\{^1H\}$ NMR (CD_2Cl_2): δ_P 11.53.

Preparation of [{(η^5-C$_5$H$_5$)(OC)$_2$Mn(μ$_2$-B*t*Bu)Cu}$_2${(η^5-C$_5$H$_5$)(OC)$_2$Mn}][B(C$_6$H$_3$Cl$_2$)$_4$] (11) and [{(*η*^{5}-C$_5$H$_5$)(OC)$_2$Mn}$_2${μ$_{\square}$-B*t*Bu}$_2$Cu][B(C$_6$H$_3$Cl$_2$)$_4$] (10)

[{(η^5-C$_5$H$_5$)(OC)$_2$Mn(μ-B*t*Bu)Cu}$_2${(η^5-C$_5$H$_5$)(OC)$_2$Mn}][B(C$_6$H$_3$Cl$_2$)$_4$] (11) In a glovebox charged with an Ar atmosphere, a solid mixture of one equivalents of the borylene complex [(η^5-C$_5$H$_5$)(OC)$_2$Mn(B*t*Bu)] (**1**) (10 mg, 0.041 mmol), one equivalent of CuCl(PPh$_3$) (15 mg, 0.041 mmol) and one equivalent of Na[BAr$^{Cl}_4$] (Ar^{Cl} = $C_6H_3Cl_2$, 25 mg, 0.041 mmol) was dissolved in ca. 5 mL of toluene. The resulting cream-colored slurry was shaken by hand for 2 minutes and a light orange cloudy solution was obtained and filtered immediately through a cotton plug. The clear orange filtrate (usually shows signs of decomposition before the filtration finishes) was collected in a small vial and stored at −30 °C for 4 days. A small amount of orange crystals formed in the vial (non-crystalline orange-brown precipitates were also present), which consists of the title compound. The orange crystals are sparingly soluble in benzene or toluene. They are moderately soluble in dichloromethane though decompose rapidly in solution. The NMR experiments of the reaction mixture and crystals were carried out though no useful information could be extracted from the spectra: multiple hydrides were observed from the reaction mixture and no signals were detected from a dichloromethane solution of the crystals. As the amount of crystals isolated is very small and highly unstable, X-ray crystallography was the only possible method of characterization (See X-ray determinations). The metal framework shows two identical moieties [Cu(μ-BtBu){Mn(η^5-C$_5$H$_5$)(CO)$_2$}] connected to an extra manganese fragment, with only one borate as a counteranion. To the best of our chemical knowledge, it seems plausible (in terms of charge and electron counting) that a hydride should be present in the structure, which makes **11** a diamagnetic compound.

[{(*η*^{5}-C$_5$H$_5$)(OC)$_2$Mn}$_2${μ$_2$-B*t*Bu}$_2$Cu][B(C$_6$H$_3$Cl$_2$)$_4$] (10) The synthesis of **10** is the same as that of **11** with an additional equivalent of cymantrene [(η^5-C$_5$H$_5$)Mn(CO)$_3$] added to the reaction mixture. The crystals formed in the cold filtered reaction mixture are **10**. Similar to the case of **11**, X-ray crystallography was the only possible method for characterization as the compound is highly sensitive and thermally unstable and can be only obtained in poor yields.

X-ray Crystallographic Determination

The crystal data of **5a–5c** and **12** were collected on a *BRUKER X8-APEX 2* (*APEX*[2] CCD-detector, *NONIUS FR-591* rotating anode generator) and those of **8–11** on a *BRUKER D8-QUEST* (*PHOTON* CMOS-detector, *INCOATEC IμS* microfocus source) diffractometer with multi-layer mirror monochromated $Mo_{K\alpha}$ radiation. The structures were solved using the intrinsic phasing method (*SHELXT*), expanded using Fourier techniques and refined with the *SHELXL* software package (see CIF files for detail on software versions).[4] All non-hydrogen atoms were refined anisotropically. Hydrogen atoms were assigned to idealized geometric positions and included in structure factor calculations. Additional details on refinement can be found in CIF files (*_refine_special_details* section). The *SHELXL* was interfaced with *SHELXLE* GUI for most of refinement steps.[5] The pictures of molecules were prepared using *POV-RAY* 3.6.2.[6] Crystallographic data can be obtained from The Cambridge Crystallographic Data Centre *via* www.ccdc.cam.ac.uk/data_request/cif

It should be mentioned that because of extensive use of residues in *SHELXL* refinement, the labels of atoms in the paper are differs from those in CIF files.

Crystal data for **5a**:

$C_{35}H_{34}AuBMnO_2P$, M_r = 780.31, yellow plate, 0.31×0.21×0.07 mm^3, triclinic space group *P*-1, a = 10.071(4) Å, b = 11.844(4) Å, c = 13.400(5) Å, α = 86.394(11)°, β = 79.387(18)°, γ = 74.371(12)°, V = 1512.9(10) Å^3, Z = 2, ρ_{calcd} = 1.713 g·cm^{-3}, μ = 5.344 mm^{-1}, F(000) = 768, T = 100(2) K, R_1 = 0.0161, wR^2 = 0.0377, 6191 independent reflections [2θ≤52.74°] and 373 parameters, CCDC-1033284.

Crystal data for **5b**:

$C_{43}H_{40}AuBMnO_2P$, M_r = 882.43, orange plate, 0.197×0.116×0.01 mm^3, monoclinic space group *C*2/*c*, a = 41.848(8) Å, b = 10.898(2) Å, c = 15.941(3) Å, β = 91.024(9)°, V = 7269(2) Å^3, Z = 8, ρ_{calcd} = 1.613 g·cm^{-3}, μ = 4.460 mm^{-1}, F(000) = 3504, T = 100(2) K, R_1 = 0.0790, wR^2 = 0.1043, 7172 independent reflections [2θ≤52.044°] and 445 parameters, CCDC-1033285.

Crystal data for **5c**:

$C_{30}H_{29}AuBMnNO_2PS$, M_r = 761.29, orange block, 0.24×0.23×0.18 mm^3, monoclinic space group $P2_1/n$, a = 13.491(2) Å, b = 13.559(2) Å, c = 16.412(2) Å, β = 101.959(15)°, V = 2937.1(8) Å^3, Z = 4, ρ_{calcd} = 1.722 g·cm^{-3}, μ = 5.572 mm^{-1}, F(000) = 1488, T = 100(2) K, R_1 = 0.0370, wR^2 = 0.0688, 6738 independent reflections [2θ≤54.998°] and 346 parameters, CCDC-1033286.

Crystal data for **8**:

The quality of the crystals of compound **8** was relatively low. The reflections were diffused and at higher resolutions (0.95–0.8 Å) smudging led to lower completeness (*e.g.* 5–10% of reflections were not integrated due to exceeding of the image queue). As observed in the case of a few earlier cymantrene-*tert*-butylborylene derivatives, because of similar volume, the B*t*Bu and cymantrene moieties can exchange their positions. The second cation in the structure was affected by this kind of disorder (see Fig. 1). This led to high residual density near the gold atom. From the remaining residual peaks only manganese and oxygen atoms could be easily localized. The bulk of the light atoms give rise to a random, diffused pattern around the borylene moiety. For this reason the minor disordered part was fitted as a rigid fragment using coordinates from the non-disordered first molecule and all its atoms, apart from the gold atom, were refined isotropically with common U_{eq} parameter. The refinement of this disorder gave an occupancy of 4% for the minor fragment.

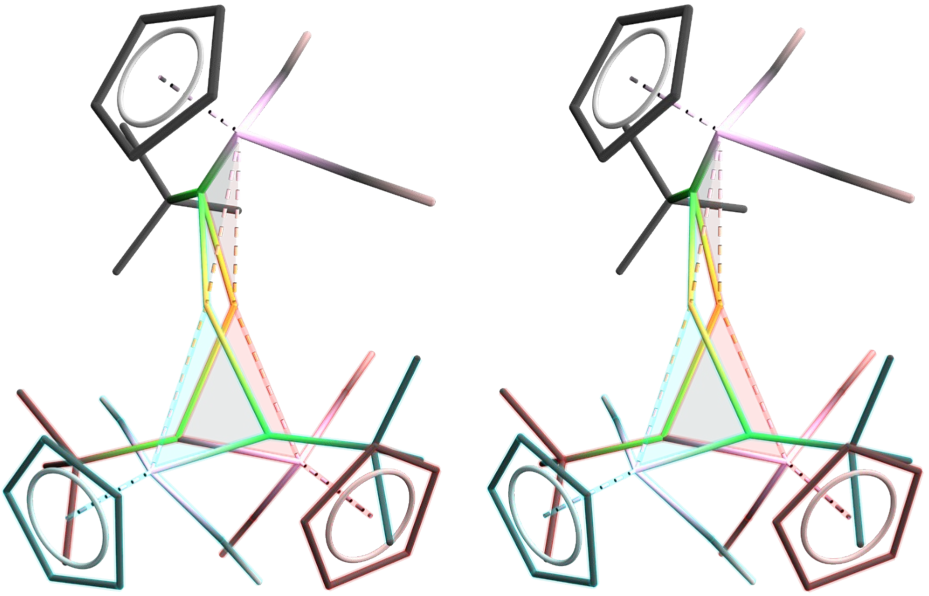

Figure S1 POV-Ray stereo-pair depiction of the disorder of the $[Au\{Cp(OC)_2Mn{=}BtBu\}_2]^+$ cation in the structure of $[\mathbf{8}]^+$. The disordered parts of molecule were overlapped with red (96%) and blue (4%) transparency.

$C_{46}H_{40}AuB_3Cl_8Mn_2O_4$, M_r = 1279.65, yellow block, 0.357×0.226×0.11 mm^3, monoclinic space group $P2_1/c$, a = 18.435(6) Å, b = 33.798(15) Å, c = 17.288(5) Å, β = 113.832(18)°, V = 9853(6) Å^3, Z = 8, ρ_{calcd} = 1.725 g·cm^{-3}, μ = 3.951 mm^{-1}, F(000) = 5024, T = 100(2) K, R_1 = 0.0626, wR^2 = 0.0999, 17575 independent reflections [$2\theta \leq 50.7°$] and 1232 parameters, CCDC-1033287.

Crystal data for **9** (**coplanar**):

$C_{98}H_{85}Ag_2B_6Cl_{16}FMn_4O_8$, M_r = 2477.21, orange plate, 0.220×0.105×0.035 mm^3, triclinic space group *P*-1, *a* = 12.2946(6) Å, *b* = 13.8214(6) Å, *c* = 16.0619(8) Å, α = 88.083(2)°, β = 67.993(2)°, γ = 83.916(2)°, *V* = 2516.2(2) Å^3, *Z* = 1, ρ_{calcd} = 1.635 g·cm^{-3}, μ = 1.350 mm^{-1}, *F*(000) = 1242, *T* = 100(2) K, R_1 = 0.0690, wR^2 = 0.1555, 10246 independent reflections [2θ≤52.742°] and 665 parameters, CCDC-1033288.

Crystal data for **9'** (**staggered**):

Compound **9** crystallized from toluene as thin plates (thinner than 0.015 Å). In polarized light these plates showed non-homogeneous color, which could indicate twinning. Indeed during the refinement, very high values of *K* for the low-intensity reflexes (*K* = Mean[F_o^2] / Mean[F_c^2]) were reported, which usually indicates twinning or a split crystal. Attempts to indicate additional domains were inconclusive. For this reason we used the standard non-twin integration procedure. All reported restraints were used on atoms belonging to the triple-disordered solvent molecule (toluene).

$C_{53}H_{48}AgB_3Cl_8Mn_2O_4$, M_r = 1282.69, colorless plate, 0.175×0.095×0.015 mm^3, monoclinic space group $P2_1/n$, *a* = 11.184(4) Å, *b* = 36.556(15) Å, *c* = 13.609(4) Å, β = 93.434(10)°, *V* = 5554(4) Å^3, *Z* = 4, ρ_{calcd} = 1.534 g·cm^{-3}, μ = 1.225 mm^{-1}, *F*(000) = 2584, *T* = 100(2) K, R_1 = 0.1203, wR^2 = 0.1631, 10554 independent reflections [2θ≤51.362°] and 730 parameters, CCDC-1033289.

Crystal data for **9** (**staggered**):

Both cations $[Ag\{Cp(OC)_2Mn{=}BtBu\}_2]^+$ in the structure of **9** (**staggered**) have shown disorder in one of cymantrene-borylene moieties. All displacement parameters of disordered atoms were restrained to the same value with similarity restraint SIMU. To keep their behavior approximately isotropic the ISOR keyword was used. The 'rigid bond' restraint was used for all bonds in the disordered parts (see the Shlexl-file included in CIF for details).

$C_{92}H_{80}Ag_2B_6Cl_{16}Mn_4O_8$, M_r = 2381.12, colorless plate, 0.216×0.139×0.084 mm^3, monoclinic space group $P2_1/c$, *a* = 18.444(9) Å, *b* = 33.901(13) Å, *c* = 17.285(5) Å, β = 113.909(17)°, *V* = 9881(7) Å^3, *Z* = 4, ρ_{calcd} = 1.601 g·cm^{-3}, μ = 1.371 mm^{-1}, *F*(000) = 4768, *T* = 100(2) K, R_1 = 0.0628, wR^2 = 0.1189, 20207 independent reflections [2θ≤52.742°] and 1500 parameters, CCDC-1033290.

Crystal data for **10**:

$C_{53}H_{48}B_3Cl_8CuMn_2O_4$, M_r = 1238.36, yellow plate, 0.165×0.158×0.074 mm^3, monoclinic space group $P2_1/n$, *a* = 11.0743(18) Å, *b* = 36.198(9) Å, *c* = 13.711(4) Å, β = 93.21(2)°, *V* = 5487(2) Å^3, *Z* = 4, ρ_{calcd} = 1.499 g·cm^{-3}, μ = 1.271 mm^{-1}, *F*(000) = 2512, *T* = 100(2) K,

R_1 = 0.0821, wR^2 = 0.1370, 11212 independent reflections [2θ≤52.742°] and 648 parameters, CCDC-1033291.

Crystal data for **11**:

The metal framework shows two identical moieties [Cu(μ-BtBu){Mn(η^5-C_5H_5)$(CO)_2$}] connected to an extra manganese fragment, with only one borate as a counteranion. To the best of our chemical knowledge, it seems plausible (in terms of charge and electron counting) that a hydride should be present in the structure, which makes **11** a paramagnetic compound (and hence the absence of the NMR signals). The highest Q peak of the structure is located next to one copper atom, which has been attributed to the previously observed disorder of the [(η^5-C_5H_5)$(OC)_2$MnM(μ-B*t*Bu)] moiety (also see Structure of **8**).[7] The next highest Q peak has been assumed for the hydride and its position was refined. It is located on the top of $MnCu_2$ ring, closer to the connecting manganese atom than to either copper atom, which complies with the overall symmetrical structure of the metal framework as well as the well-known [(η^5-C_5H_5)$(CO)_2$MnH]$^-$ anion.

$[C_{29}H_{34}B_2Cu_2Mn_3O_6][C_{24}H_{12}BCl_8]\times2(CH_2Cl_2)$, M_r = 1556.68, orange plate, 0.15×0.14×0.05 mm^3, triclinic space group *P*-1, *a* = 11.952(4) Å, *b* = 14.576(5) Å, *c* = 20.573(6) Å, α = 70.672(14)°, β = 73.210(17)°, γ = 68.233(18)°, *V* = 3084.0(17) Å^3, *Z* = 2, ρ_{calcd} = 1.676 g·cm^{-3}, μ = 1.844 mm^{-1}, *F*(000) = 1560, *T* = 100(2) K, R_1 = 0.0391, wR^2 = 0.0790, 12602 independent reflections [2θ≤52.742°] and 739 parameters, CCDC-1033292.

Crystal data for **11'**:

The geometry of the minor part of disordered $[C_{29}H_{34}B_2Cu_2Mn_3O_6]^-$ was fitted to the geometry of the dominant residue. The minor residue was refined isotropically. Also, the solvent molecules (toluene) were fitted to the idealized geometry.

$[C_{29}H_{34}B_2Cu_2Mn_3O_6][C_{24}H_{12}BCl_8]\times2(C_7H_8)$, M_r = 1571.09, yellow plate, 0.322×0.098×0.02 mm^3, Triclinic space group *P*-1, *a* = 11.301(4) Å, *b* = 14.461(3) Å, *c* = 21.311(7) Å, α = 97.172(14)°, β = 96.19(3)°, γ = 102.139(18)°, *V* = 3345.5(17) Å^3, *Z* = 2, ρ_{calcd} = 1.560 g·cm^{-3}, μ = 1.546 mm^{-1}, *F*(000) = 1592, *T* = 100(2) K, R_1 = 0.0690, wR^2 = 0.1408, 15988 independent reflections [2θ≤55.836°] and 858 parameters, CCDC-1033293.

Crystal data for **12**:

Some of the CF_3 groups in this structure showed rotational disorder and were refined as disordered. The atomic displacement parameters in these groups were restricted with SIMU

and DELU restraints. The U_{ii} displacement parameters of some F atoms (see the Shelx file included in CIF) were restrained with the ISOR keyword to approximate isotropic behavior.

$C_{75}H_{50}AuBF_{24}P_2$, M_r = 1676.86, colorless plate, 0.28×0.18×0.08 mm^3, triclinic space group *P*–1, *a* = 12.380(3) Å, *b* = 15.772(5) Å, *c* = 18.327(4) Å, α = 83.065(19)°, β = 74.699(13)°, γ = 81.63(2)°, *V* = 3402.2(16) Å^3, *Z* = 2, ρ_{calcd} = 1.637 g·cm^{-3}, μ = 2.319 mm^{-1}, *F*(000) = 1660, *T* = 100(2) K, R_1 = 0.0647, wR^2 = 0.1105, 13833 independent reflections [2θ≤52.744°] and 1037 parameters, CCDC-1033294.

Computational Details Section

General considerations

The gas-phase geometry pre-optimizations were performed using *TURBOMOLE 6.5*.[8] The final optimizations and the preparation of wave-function files were performed using the *GAUSSIAN 09* program[9] or the *ADF 2013* package.[10,11] The B3LYP hybrid functional and respectively Def2-SVP[12] or TZ2P basis set were used for all these computations.[13] We ensured that the calculated geometries are respectively minima on the potential energy surface by carrying out harmonic frequency calculations (zero negative eigenvalues of the Hessian). The Wiberg Bond Index (WBI) values given in the text as well as the Natural Bond Orbital analysis were obtained using the *NBO 6* program.[14] The Electron Localization Function (ELF) was computed by employing the *TOPMOD* package[15] using wave-functions obtained with *GAUSSIAN 09*. Illustrations of the NBO orbitals and ELF were prepared with the *MOLEKEL 4.3*.[16] The Energy Decomposition Analysis (EDA), the fragment orbital (FO) computations, as well as ETS-NOCV decomposition were calculated within the Zero Order Regular Approximation (ZORA) formalism at the B3LYP/TZ2P level with *ADF 2013*. The pictures of fragment orbitals (SFOs) were prepared using *ADF-GUI*.[17] The topology of $\nabla^2\rho$ (*Atoms in Molecules*) based on Def2-SVP basis was calculated with *AIMALL* program (ver. 13.11.04).[18] Quantitatively equivalent results for the TZ2P basis were obtained with ADF package.

Natural Bond Orbital Analysis (NBO) for cation [10]$^+$

The manganese AOs (*sd*$^{2.4}$ hybrid) contribute 40% of the two-center Mn–B bond (BD), while boron AOs (*sp*$^{1.24}$ hybrid) account for the remaining 60%. This orbital was found in second order perturbation theory analysis to interact with low-populated lone vacant orbital (LV) of the copper atom (ΔE_{int} = 145.1 kcal·mol^{-1}). The graphic interpretation of this dative interaction is presented in Figure S2. As expected for a molecule with C_2 symmetry, two of this kind of interaction could be found.

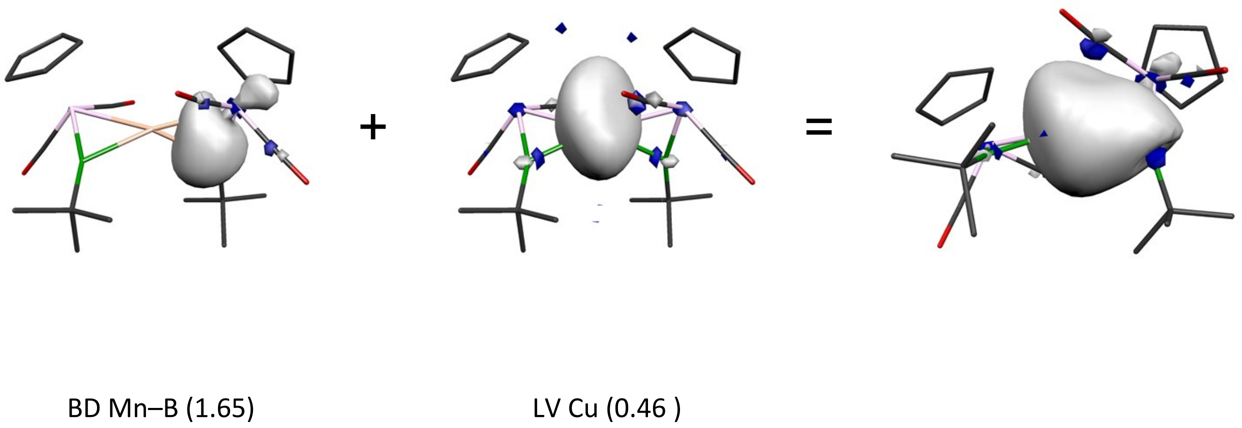

Figure S2. Interaction between the two-center bond Mn–B and the anti-bonding one-center lone vacant orbital of the copper atom, and their resulting three-center orbital. The numbers in brackets are electron occupancies of orbitals.

The second-strongest interaction (ΔE_{int} = 24.4 kcal·mol^{-1}), shown in Figure S3, comes about through the donation from two-center Mn–C orbital (26% of sd$^{1.7}$ hybrid of manganese and 74% of sp$^{0.5}$ of carbon) into the same lone vacant orbital of the copper atom. This interaction emphasizes impact of the carbonyl ligand on the stability of the cation **10$^+$**.

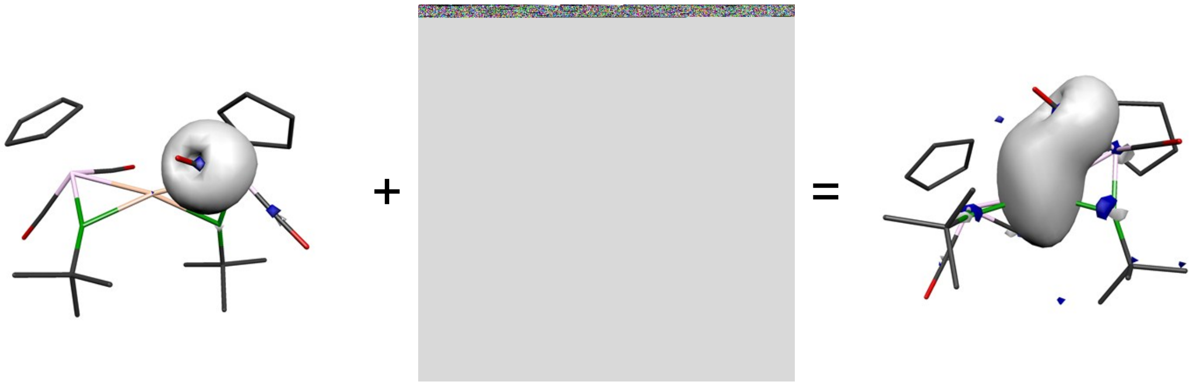

BD Mn–C (1.92)　　　　LV Cu (0.46)

Figure S3. Interaction between the manganese-carbonyl moiety and the copper atom and their resulting two-center orbital. The numbers in brackets are electron occupancies of orbitals. The labeling of the gold-centered pair as anti-bonding and manganese-centered as bonding is arbitrary in NBO software. As the numbers show, both have very similar population.

The back-bonding from copper to borylene moieties, shown in Figure S4, is ten-fold weaker (ΔE_{int} = 9.7 kcal·mol^{-1}) than the bonding of borylene to Cu^{+}. This interaction is described by donation from the copper core pair (CR) into low-populated Mn–B two-center valence antibond (BD*).

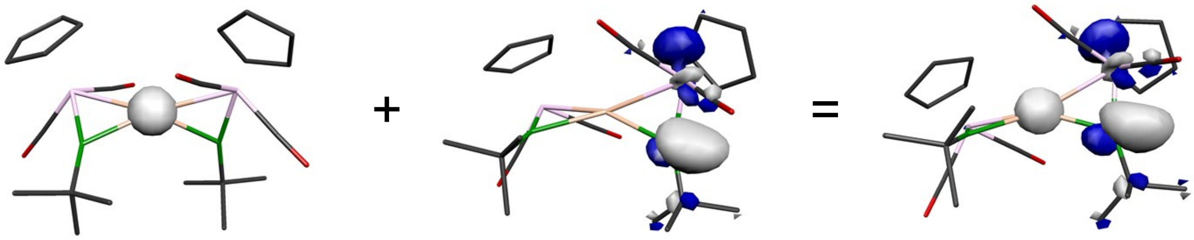

CR Cu(2.0)　　　　BD* Mn–C (0.25)

Figure S4. Weak back-bonding interaction between the copper atom and the antibonding Mn–B orbital and resulting orbital. The numbers in brackets are electron occupancies of orbitals. The labeling of the Mn–C pair as anti-bonding and copper-centered as core is arbitrary in NBO software.

Electron Localization Function (ELF) Topology for cation $[10]^+$

The ELF topology of the CuMnB rings is determined by three synaptic basins that have population of ~2.4 ē. Adjacent to these basins along B–Mn bonds, outside of the rings, are located smaller basins with populations of ~0.8 ē (see Figure S5). These sets of valence basis create envelopes that in form are very similar to that calculated earlier for $[Cp(OC)_2Mn(BClMe)][Au(PMe_3)]$.

Table 1. Volumes [$Bohr^3$] and populations of valence ELF basins in CuMnB rings. A graphical representation is shown in Figure S5.

	Basin	Volume	Population
1	V(Cu,Mn1,B1)	81.10	2.42
2	V(Cu,Mn1,B1)	80.90	2.39
3	V(Mn1,B1)	20.04	0.80
4	V(Mn2,B2)	19.79	0.77

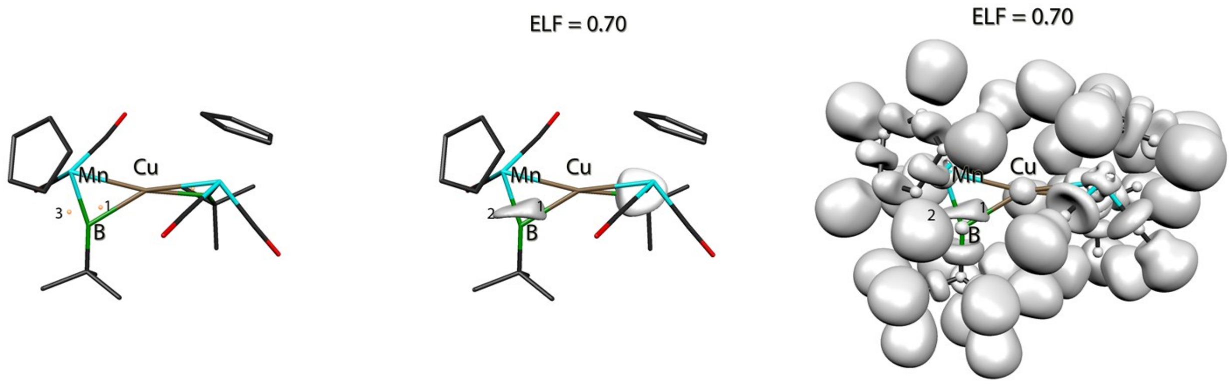

Figure S5. Location of ELF attractors (left, numeration as in table) and the *iso*-surface of Electron Localization Function in [**10**]$^+$ at 0.75 *iso*-value (middle). For clarity only envelopes of two synaptic valence basins describing an interaction in the CuMnB ring are shown. The picture on the right side shows unpruned ELF = 0.75.

Energy Decomposition Analysis (EDA) of $[8]^+$–$[10]^+$

For all three complexes the electrostatic gain is balanced by Pauli repulsion ($\Delta E_{\mathrm{ElStat}}$ vs. $\Delta E_{\mathrm{Pauli}}$). Roughly 1/3rd of the constructive interaction comes from orbital interaction ΔE_{Orb}. The rest is caused by electrostatic interaction $\Delta E_{\mathrm{ElStat}}$ between the borylene [Cp(OC)$_2$Mn=B*t*Bu] and the coinage metal cation M^+.

Table 2. Components of the interaction Energy (ΔE_{Int}) and Bond Dissociation (D_0) [kcal·mol^{-1}] for cationic compounds **[8]$^+$–[10]$^+$**. $\Delta E_{\mathrm{Steric}} = \Delta E_{\mathrm{ElStat}} + \Delta E_{\mathrm{Pauli}}$; $\Delta E_{\mathrm{Int}} = \Delta E_{\mathrm{Steric}} + \Delta E_{\mathrm{Orb}} = \Delta E_{\mathrm{ElStat}} + \Delta E_{\mathrm{Pauli}} + E_{\mathrm{Orb}}$; E_{SP} is a single-point energy in final geometry; E_{Opt} is an energy after geometry optimization of the fragment; bond dissociation energy (D_0) is defined as $D_0 = \Delta E_{Int} + \Sigma\Delta E_{Prep}$, where preparation energy of a fragment (ΔE_{Prep}) is defined as: $\Delta E_{Prep} = E_{\mathrm{SP}} - E_{\mathrm{Opt}}$. Note that two borylene fragments (*e.g.* Fragments 2) have to be considered.

	**[8]$^+$** (M = Au)	**[9]$^+$** (M = Ag)	**[10]$^+$** (M = Cu)
$\Delta E_{\mathrm{ElStat}}$	–301.08	–181.62	–192.07
ΔE_{Orb}	–176.53	–106.23	–125.36
$\Delta E_{\mathrm{Pauli}}$	301.28	172.20	174.35
$\Delta E_{\mathrm{Steric}}$	–0.20	–9.42	–17.72
ΔE_{Int}	–176.33	–115.65	–143.08
$\Delta E_{\mathrm{Orb}} / \Delta E_{\mathrm{Int}}$	100%	92%	88%
$\Delta E_{\mathrm{Orb}} / (\Delta E_{\mathrm{Orb}} + \Delta E_{\mathrm{ElStat}})$	37%	37%	39%

	[8]$^+$	[9]$^+$	[10]$^+$
E_{SP} (Frag1†) = E_{Opt}	203.01	171.72	182.44
E_{SP} (Frag2‡)	–4662.89	–4665.91	–4667.06
E_{Opt} (Frag2)	–4703.20	–4703.20	–4703.20
ΔE_{Prep} (Frag1)	0	0	0
ΔE_{Prep} (Frag2)	40.31	37.30	36.15
D_0	–95.71	–41.06	–70.79

† Frag1 = M^+ (M = Cu, Ag, Au)
‡ Frag2 = [(η^5-C_5H_5)(OC)$_2$Mn=B*t*Bu]$^+$

Charges $[8]^+$–$[10]^+$

The following tables list the charges obtained from different types of analyses.

Table 3. Charges computed for cationic compounds **$[8]^+$–$[10]^+$** (M = Cu, Ag, Au). All results based on ADF calculations at B3LYP/ TZ2P(sc) level.

	**$[8]^+$** (M = Au)	**$[9]^+$** (M = Ag)	**$[10]^+$** (M = Cu)
Voronoi charge			
M^+	0.683	0.591	0.341
[Cp(OC)$_2$Mn=B*t*Bu]	0.159	0.204	0.330
Voronoi Deformation density			
M^+	–0.255	–0.220	–0.291
[Cp(OC)$_2$Mn=B*t*Bu]	0.128	0.110	0.146
Hirshfeld Charge			
M^+	0.726	0.796	0.826
[Cp(OC)$_2$Mn=B*t*Bu]	0.137	0.102	0.087
Natural Charge			
M	0.503	0.669	0.692
Mn	–0.560	–0.604	–0.706
B	0.667	0.746	0.731
Mulliken Charge			
M	0.058	0.294	0.028
Mn	0.489	0.396	0.490
B	0.044	0.029	0.070

Atoms in Molecules (AiM) – Topology of $\nabla^2\rho$ for cation [8]–[10]$^+$

No bond critical point (BCP) between the coinage metal cation M^+ (M = Au, Ag, Cu) and manganese, nor any ring critical point in $\nabla^2\rho$ was found for either three-membered ring [BMnM]. The only localized critical points were BCPs for the bond paths B–Mn and B–M (Figure S6). However the shape of valence shell charge concentration (VSCC) close to the boron atom closely matches that computed for [Cp(OC)$_2$Mn(BClMe)][Au(PMe$_3$)] (compared in Figure S7), and it supports the hypothesis that the interaction between borylene and coinage metal has three-center bond character, *i.e.* it comes from interaction of manganeseborylne moiety with coinage-metal (π-Mn=B → Cu) rather than from covalent interaction B–Cu.

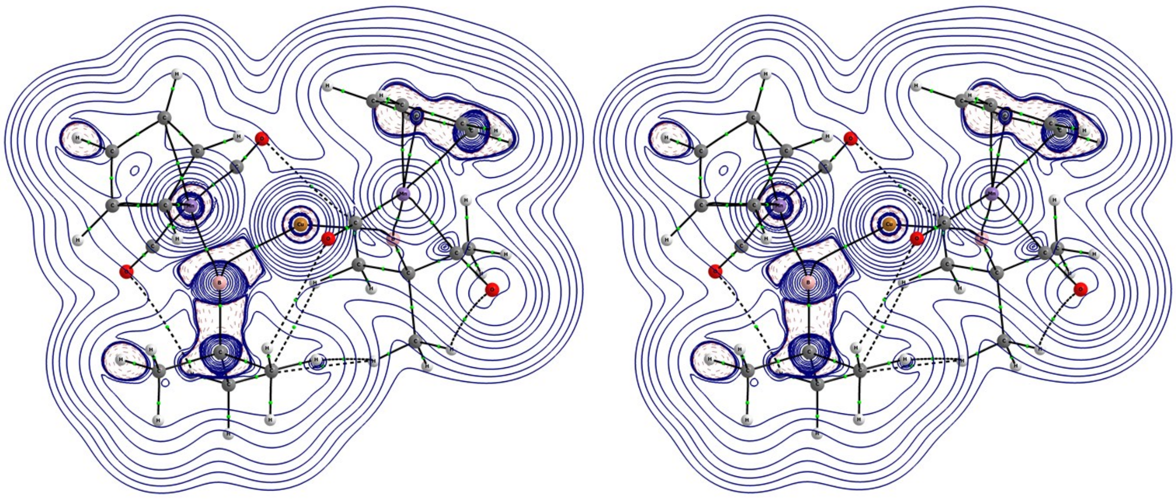

Figure S6. Laplacian of electron density $\nabla^2\rho$ and its topology in [**10**]$^+$. The contour lines are drawn in the plane defined by [MnBCu]. Black lines constitute bond paths (red lines indicate negative values in $\nabla^2\rho$, blue lines indicate positive values); green dots represent bond critical points.

It is worth mentioning that despite the fact that the second order perturbation analysis in NBO showed a weak interaction (33.0 kcal·mol^{-1}) originating from a Mn–C two-center bond to the copper atom, no such bonding path nor respective critical point were found in the topology of ρ for cation [**10**]$^+$

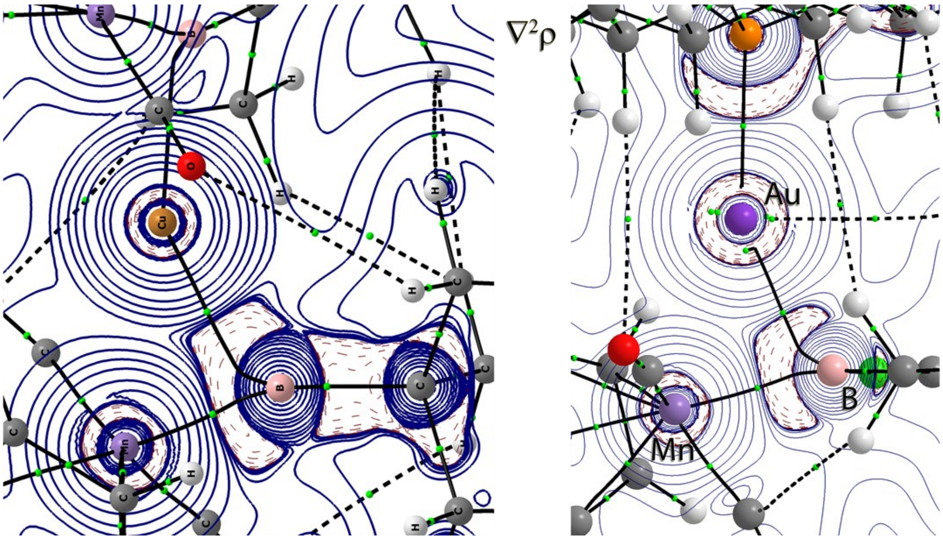

Figure S7. Comparison of Laplacian of electron density $\nabla^2\rho$ in [**10**]$^+$ (left) and [Cp(OC)$_2$Mn(BClMe)][Au(PMe$_3$)] (right). The path B–Au does not end at the center of the Au atom (right) because computations for this element were performed with effective core potential basis (ECP).

ETS-NOCV analysis of [10]+

The following figures 8 and 9 and table illustrate the fragment orbitals used for the most significant ETS-NOCV interaction.

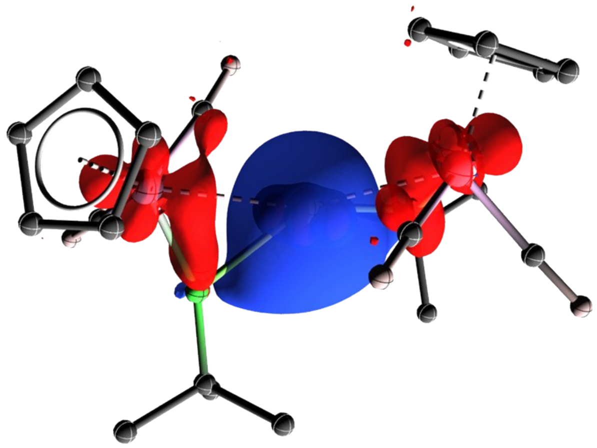

Figure S8. Deformation density with the strongest interaction in [**10**]+.

Table 4. The SFO contributions to NOCV interaction presented in Figure S8. The fragment label “MnB” is used for borylene [Cp(OC)$_2$Mn=B(*t*Bu)].

Fragment	SFO index in [**10**]+	SFO index in fragment	Label in fragment	SFO contribution
Cu+	15	2 *s*	LUMO	0.31620
MnB(1)	112	63	HOMO	–0.17789
MnB(2)	668	63	HOMO	–0.17722
Cu+	19	3 *s*	LUMO+4	0.05820
MnB(1)	113	64	LUMO	0.03557
MnB(2)	669	64	LUMO	0.03538
Cu+	13	1 D:xy	HOMO†	–0.02934
MnB(2)	666	61	HOMO–2	–0.01323
MnB(1)	110	61	HOMO–2	–0.01292

† This orbital is fivefold degenerate, *e.g.* the numbering is HOMO–4 — HOMO.

MnB 61 (HOMO–2) → Cu^+ *s* (LUMO, LUMO+4)

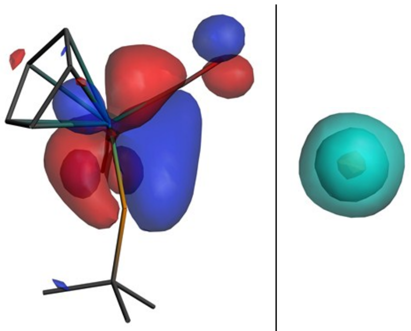

MnB 63 (HOMO) → Cu^+ *s* (LUMO, LUMO+4)

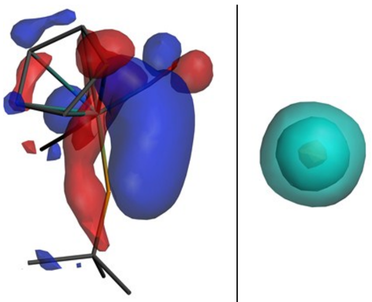

MnB 64 (LUMO–2) ← Cu^+ 30 (LUMO)

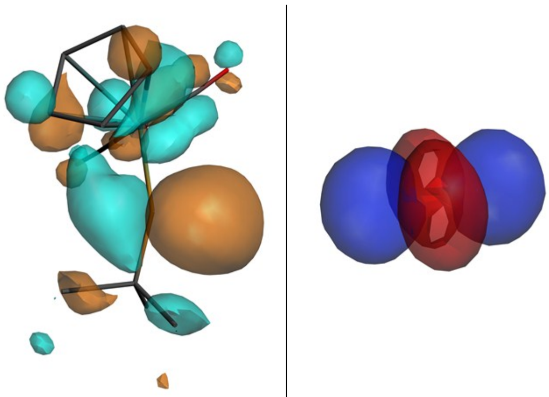

Figure S9. Orbitals from fragments [Cp(OC)$_2$Mn=B(*t*Bu)] and Cu^+ representing π-MnB → s^*-Cu^+ interaction and d_{xy} Cu^+ back donation.

Comparison of ETS-NOCV results for [8]⁺–[10]⁺

The following Figure 10 illustrate first four deformations densities with the highest NOCV eigenvalue for cations **[8]⁺–[10]⁺**. Shapes of contours is similar for all three coinage metal complexes, however in over all the contours are smaller for **[9]⁺**.The orbital interaction energies match closely bond dissociation energies (D_0): the strongest interaction is found for gold and the weakest for the silver complex.

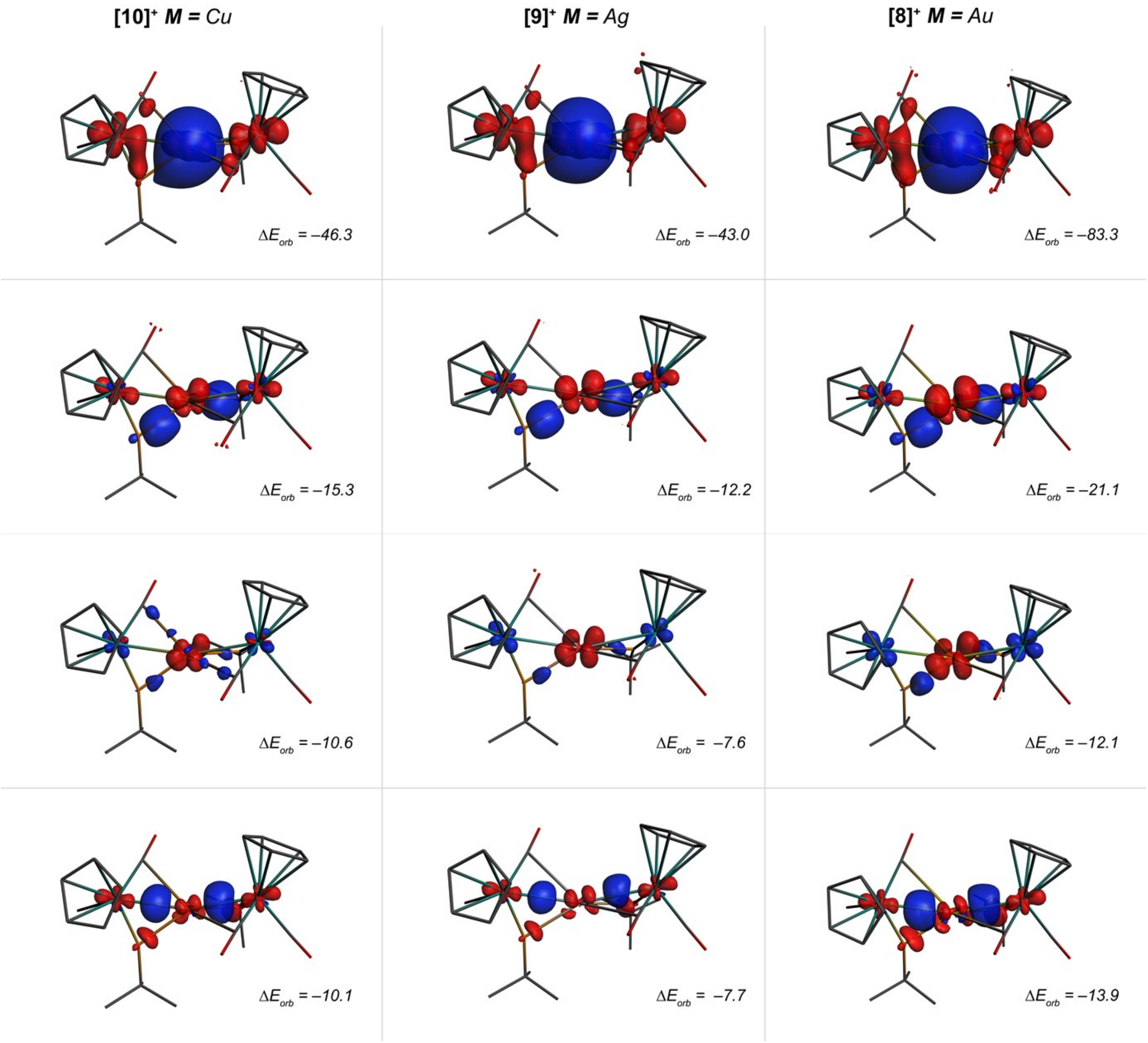

Figure S10. Deformation densities in **[8]⁺–[10]⁺** with corresponding orbital interactions energies [kcal·mol^{-1}]. The *iso*-value used for this graphic was set to 0.0025 a.u. The red coloration indicates electron density depletion, blue concentration..

Calculated and experimental IR-spectra of [11]$^+$

We have computed the structure of **[11]$^+$** using def-SV(P)/B3LYP level of theory. The additional hydrogen was initially placed at the position used in X-Ray refinement. The optimized geometry has retained this hydrogen at its original localization (Figure 11). Simulated, non-scaled IR-Spectrum of **[11]$^+$** is shown at Figure 12. The vibration of hydrogen was predicted to be at 1237.6 cm^{-1} with relative intensity of 4% to the highest peak in spectrum. The strong CO-stretches are calculated in range of 2097–1993 cm^{-1} as compared to observed in IR-spectrum from solid sample of 1980–1840 cm^{-1} (Figure S13). Applying these strong vibrations for calculation of scaling factor (0.938), moves the H-vibration to 1160.9 cm^{-1}. The experimental 1151.3 cm^{-1} band is very close to this value, however, as this peak is too close to the baseline noise, it cannot be unambiguous proof for presence of this hydrogen.

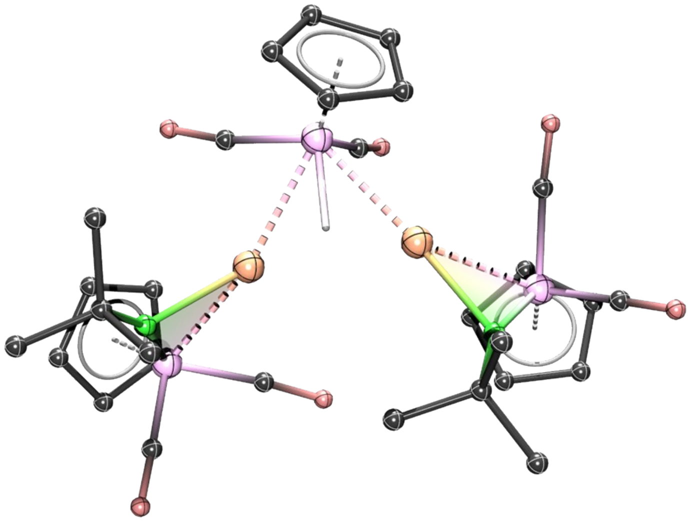

Figure S11. Computed geometry of **[11]$^+$**.

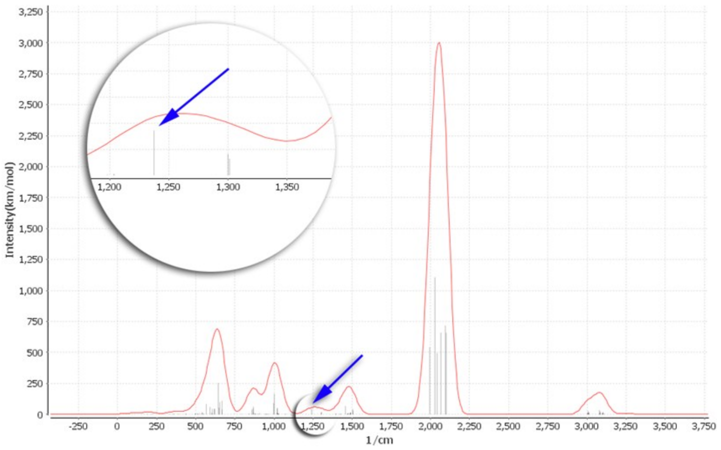

Figure S12. IR spectrum of **[11]⁺**. The metal-bond-H vibration at 1237.6 cm^{-1} is marked with arrow (relative intensity ~4%).

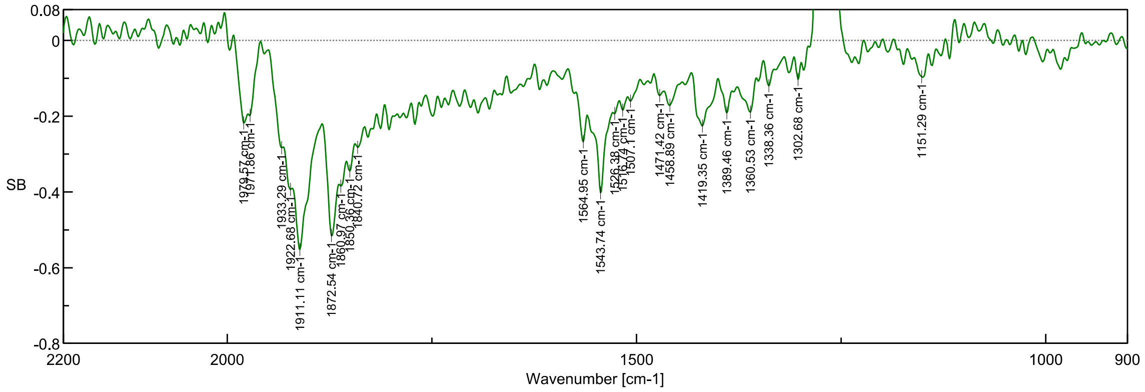

[Comments]
Sample name
Comment
User
Division
Company Universität Würzburg

[Detailed Information]
Creation date 23.02.2015 11:42

Data array type Linear data array
Horizontal axis Wavenumber [cm-1]
Vertical axis SB
Start 399.193 cm-1
End 6500.86 cm-1
Data interval 0.964233 cm-1
Data points 6329

[Measurement Information]
Model Name FT/IR-6200typeA
Serial Number A014361022

Measurement Date 23.02.2015 11:13

Light Source Standard
Detector GloveBox HWG
Accumulation 32
Resolution 4 cm-1
Zero Filling On
Apodization Cosine
Gain Auto (2)
Aperture Auto (7.1 mm)
Scanning Speed Auto (1 mm/sec)
Filter Auto (3000 Hz)

Figure S13. Experimental solid IR spectrum of **[11]⁺**.

References

(1) Braunschweig, H.; Burzler, M.; Kupfer, T.; Radacki, K.; Seeler, F. *Angew. Chem. Int. Ed.* **2007**, *46*, 7785.

(2) Braunschweig, H.; Kramer, T.; Radacki, K.; Shang, R.; Siedler, E.; Werner, C. *Chem. Sci.* **2014**, *5*, 2271.

(3) Al-Sa'Ady, A. K.; McAuliffe, C. A.; Parish, R. V.; Sandbank, J. A.; Potts, R. A.; Schneider, W. F. *Inorganic Syntheses*; John Wiley & Sons, Inc.: 2007, p 191.

(4) Sheldrick, G. M. *Acta Crystallogr., Sect. A Found. Crystallogr.* **2008**, *A64*, 112.

(5) Huebschle, C. B.; Sheldrick, G. M.; Dittrich, B. *J. Appl. Crystallogr.* **2011**, *44*, 1281.

(6) Cason, C. *et al.*; Persistence of Vision Pty. Ltd., **2009**; http://www.povray.org/download/.

(7) Braunschweig, H.; Radacki, K.; Shang, R.; Tate, C. W. *Angew. Chem., Int. Ed.* **2013**, *52*, 729.

(8) Ahlrichs, R. *et al. Turbomole 6.5*; Turbomole GmbH: Karlsruhe (Germany); (**2013**).

(9) Frisch, M. J. *et al. Gaussian09*; *Revision D.01*; Gaussian Inc.: Wallingford CT (USA); (**2013**).

(10) van Lenthe, E.; Ehlers, A.; Baerends, E. J. *J. Chem. Phys.* **1999**, *110*, 8943.

(11) *GUI 2013*; *SCM* Amsterdam; http://www.scm.com (**2013**).

(12) Weigend, F.; Ahlrichs, R. *Phys. Chem. Chem. Phys.* **2005**, *7*, 3297.

(13) Def2-SVP basis set was obtained from the Extensible Computational Chemisty Environment Basis Set Database, Version 1.2.2, as developed and distributed by the Molecular Science Computing Facility, Environmental and Molecular Sciences Laboratory which is part of the Pacific Northwest Laboratory, P.O. Box 999, Richland, Washington 99352 (USA), and funded by the U. S. Department of Energy.

(14) Glendening, E. D.; Badenhoop, J. K.; Reed, A. E.; Carpenter, J. E.; Bohmann, J. A.; MOrales, C. M.; Weinhold, F.; *NBO 5.0*; Theoretical Institute, University of Wisconsin: Madison (USA); (**2001**).

(15) Noury, S.; Krokidis, X.; Fuster, F.; Silvi, B. *TopMoD Package*; Universite Pierre et Marie Curie: Paris (France), **1997**.

(16) Flueiger, P.; Luethi, H. P.; Portmann, S.; Weber, J. *MOLEKEL 4.0*; Swiss Center for Scientific Computing: Manno (Switzerland), **2000**.

(17) *ADF 2013*; SCM, Theoretical Chemistry, Vrije Universiteit: Amsterdam (The Netherlands), **2003**.

(18) Keith, T. A. *AIMAll*; *Version 13.05.06*; TK Gristmill Software: Overland Park KS (USA); http://aim.tkgristmill.com (**2013**).
